# Multichromosomal mitochondrial genome of *Punica granatum*: comparative evolutionary analysis and gene transformation from chloroplast genomes

**DOI:** 10.1186/s12870-023-04538-8

**Published:** 2023-10-25

**Authors:** Lijuan Feng, Zenghui Wang, Chuanzeng Wang, Xuemei Yang, Mengmeng An, Yanlei Yin

**Affiliations:** 1 Shandong Institute of Pomology, Taian, 271000 Shandong China; 2 Shandong Academy of Agricultural Sciences, Jinan, 250100 Shandong China; 3 Zibo Academy of Agricultural Sciences, Zibo, 255000 Shandong China

**Keywords:** *Punica granatum*, Mitogenome, Multi-chromosomal structure, Evolutionary relationship, Intracellular gene transfer

## Abstract

**Background:**

Punica granatum is a fundamentally important fruit tree that has important economic, medicinal and ornamental properties. At present, there are few reports on the mitochondrial genome of pomegranate. Hence, in this study the *P. granatum* mitogenome was sequenced and assembled to further understanding of organization, variation, and evolution of mitogenomes of this tree species.

**Results:**

The genome structure was multi-chromosomes with seven circular contigs, measuring 382,774 bp in length with a 45.91% GC content. It contained 74 genes, including 46 protein-coding genes, 25 tRNA genes, and three rRNA genes. There were 188 pairs of dispersed repeats with lengths of 30 or greater, primarily consisting of reverse complementary repeats. The mitogenome analysis identified 114SSRs and 466 RNA editing sites. Analyses of codon usage, nucleotide diversity and gene migration from chloroplast to mitochondrial were also conducted. The collinear and comparative analysis of mitochondrial structures between *P. granatum* and its proximal species indicated that *P. granatum* ‘Taishanhong’ was closely related to *P. granatum* ‘Qingpitian’ and *Lagerstroemia indica.* Phylogenetic examination based on the mitogenome also confirmed the evolutionary relationship.

**Conclusion:**

The results offered crucial information on the evolutionary biology of pomegranate and highlighted ways to promote the utilization of the species’ germplasm.

**Supplementary Information:**

The online version contains supplementary material available at 10.1186/s12870-023-04538-8.

## Introduction

Pomegranate (*Punica granatum* L.) belongs to the genus *Punica* of the Lythraceae family, and is an essentially valuable fruit tree in the world [1–3]. It is native to Central Asia region and grows mainly in tropical and subtropical countries such as India, Iran, China, and the USA [4, 5]. The consumption of pomegranates has shown a remarkable increase due to their exceptional economic, nutritional, medicinal and ornamental value. The different parts (fruit peel, juice, seeds, flowers, leaves, bark) are rich in bioactive constituents, and have various medicinal advantages to provide high antioxidant, antineoplastic, anticancer and phytonutrient capacity [6–9]. At present, pomegranate related industries are expanding rapidly, and plays an important role in the economic, cultural and ecological fields.

Mitochondria are semi-autonomous organelles with a mitochondrial genome found in virtually all eukaryotic cells, which synthesize Adenosine Triphosphate (ATP) through the tricarboxylic acid cycle and oxidative phosphorylation to provide energy for cells [10, 11]. Mitochondria are involved in numerous metabolic processes and perform crucial roles in cell differentiation, apoptosis, cell development, and cell division by converting biomass energy into chemical energy for daily activities [12–14]. Plant chloroplast genomes have revealed conserved genome structure, but the mitochondrial genome has very low collinearity due to extensive rearrangement and repeat sequences, whereas its protein-coding genes are relatively conserved [12, 15]. Plant mitogenomes are species specific and possess many unique features, which vary considerably in length, gene order, and gene content [16, 17]. The structures of mitogenomes are normally depicted as a complex and dynamic circular structure with a large amount of unconserved DNA of unknown function [18]. In some species, the mitochondrial genomes have linear conformations, branched and multichromosomal structures [14, 19]. The above characteristics of the mitochondrial genome provide helpful information for evolutionary and phylogenetic studies.

The mitochondrial genomes of 545 plant species have been assembled and reported [14], including Sapindaceae [17], Rosaceae [20], Anaeardiaceae [21], and Myrtaceae [22] family. Mitochondrial genomes of some fruit crops have been reported, such as *malus domestica* [23], *Satsuma mandarin* [24], and kiwifruit [25], etc. While the chloroplast genomes of approximately 114 species of *P. granatum* are available in the NCBI database (as of 28 June 2023), only one pomegranate variety’s mitochondrial genome has been identified and published [26]. The germplasm resources of pomegranate are abundant, but the mitochondrial genome differences between different varieties are still unclear. Therefore, more in-depth researches of the mitochondrial genome of *P. granatum* are of great significance for its effective utilization and genetic improvement.

In this study, we sequenced, assembled and annotated the complete mitochondrial genome of main *P. granatum* cultivar ‘Taishanhong’. The mitogenome characteristics, repetitive sequences, SSR identification, and RNA editing prediction were investigated. Further analyses regarding species synteny and phylogeny were carried out for the determination of phylogenic positions and molecular diversity of the myrtales. The gene transformation of chloroplast genome to mitochondrion DNA was analyzed. This study is anticipated to lay the foundation for a deeper understanding and exploration of evolutionary variations and genomic breeding of pomegranate mitochondrial genomes.

## Results

### Genomic features of *P. granatum* mitochondrial genome

**Fig. 1:**
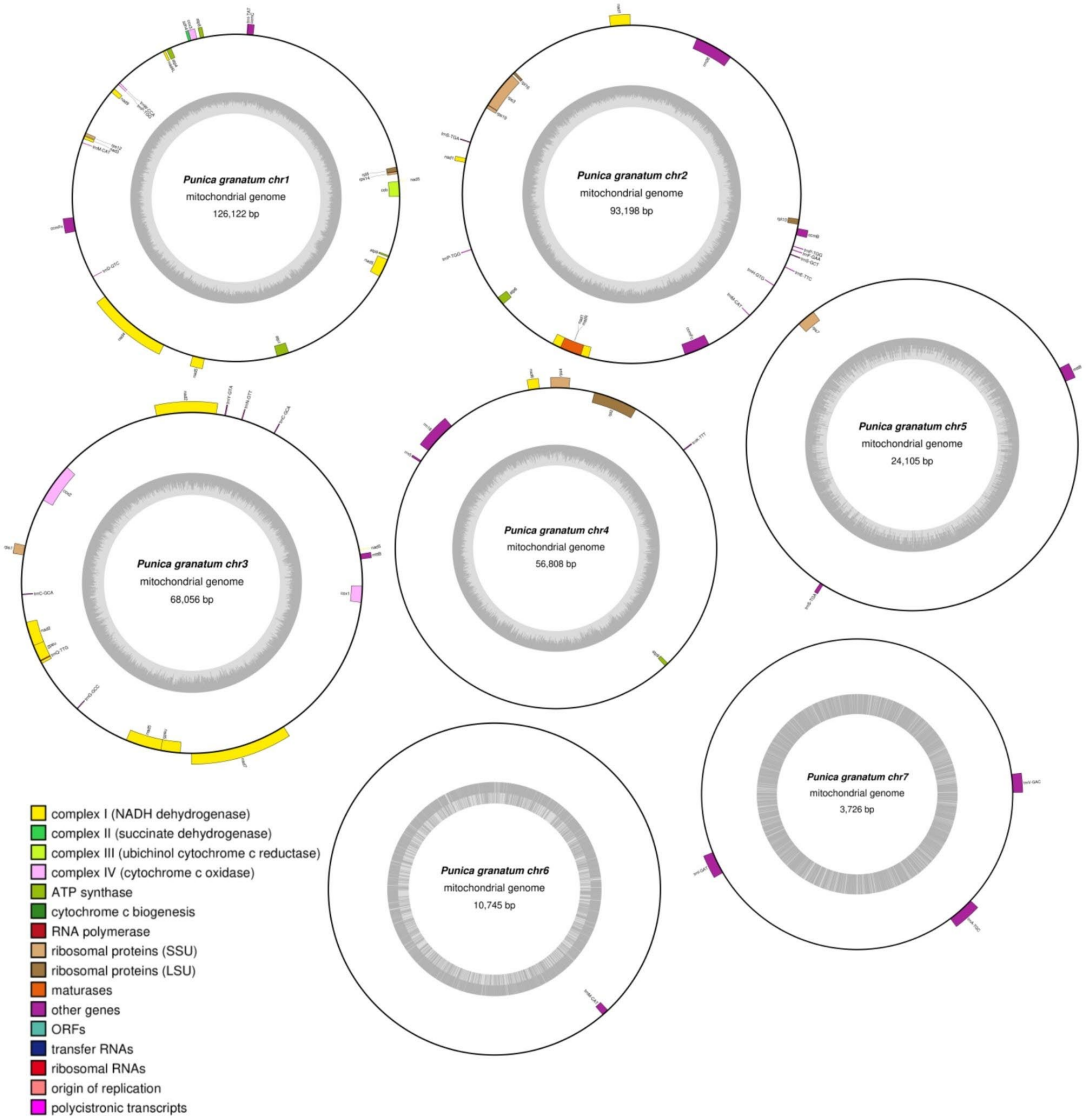
Circular Map of the *P. granatum* mitochondrial genome. The genome consisted of 7 circular chromosomes. The genes that code in the forward direction are on the outside of the circle, and the genes that code in the opposite direction are on the inside of the circle. The inside gray circle represents GC content

The *P. granatum* mitochondrial genome was sequenced, assembled, and annotated, which has been archived in GenBank under the accession numbers OQ973289, OQ973290, OQ973291, OQ973292, OQ973293, OQ973294 and OQ973295. The mitochondrial genome of *P. granatum* was assembled into 7 circular contigs with lengths ranging from 3,728 bp to 126,124 bp, with a total length of 382,774 bp (Fig. 1, Fig. S1 and Fig. S2). The total GC content was 45.91%, ranging between 45.42% and 52.60% (Table S1). It encoded 74 genes, including 46 mitochondrial protein-coding genes, 25 tRNA genes, and three rRNA genes (rrn5, rrn18, and rrn26). The total GC contents of CDS, tRNA and rRNA genes were 42.35%, 50.85% and 52.14%, respectively (Table S1).

The protein-coding genes included 34 core genes and nine variable genes (Table 1). The core genes included five ATP synthase genes (atp1, atp4, atp6, atp8 and atp9), nine NADH dehydrogenase genes (*nad1*, *nad2*, *nad3*, *nad4*, *nad4L*, *nad5*, *nad6*, *nad7* and *nad9*), four cytochrome c biogenesis genes (*ccmB*, *ccmC*, *ccmFC* and *ccmFN*), three cytochrome c oxidase genes (*cox1*, *cox2* and *cox3*), one Maturases gene (*matR*), one membrane transport protein gene (*mttB*) and one ubichinol cytochrome c reductase gene (*cob*). The variable genes included four large subunits of ribosomal protein (*rpl10*, *rpl16*, *rpl2* and *rpl5*), seven small subunits of ribosomal protein (*rps1*, *rps3*, *rps4*, *rps7*, *rps12*, *rps14* and *rps19*), and one succinate dehydrogenase (*sdh4*). Among these, the genes *atp9*, *mttB*, *nad2* and *nad5* had two copies. The *nad1*, *nad2*, *nad5* and *nad7* genes contained four introns and *nad4* gene had three introns, while *ccmFC*, *cox2*, *rpl2* and *rps3* had one intron. The *trnC-GCA*, *trnM-CAT*, *trnP-TGG* and *trnS-TGA* genes were multi-copy genes. The *trnA-TGC*, *trnI-GAT* and *trnI-TAT* genes contained one intron.

**Table 1:** Gene composition in the mitogenome of *P. granatum*

Group of Genes	Gene name	Length	Start codon	Stop codon	Amino acid
ATP synthase	atp1	1536	ATG	TAA	512
	atp4	597	ATG	TAG	199
	atp6	747	ATG	TGA	249
	atp8	480	ATG	TAA	160
	atp9(2)	225	ATG	CGA(TGA)	75
Cytohrome c biogenesis	ccmB	621	ATG	TGA	207
	ccmC	753	ATG	TGA	251
	ccmFc	1356	ATG	TAA	452
	ccmFn*	1737	ATG	TAA	579
Ubichinol cytochrome c reductase	cob	1890	ATG	TAA	630
Cytochrome c oxidase	cox1	1065	ATG	TAA	355
	cox2*	783	ATG	TAA	261
	cox3	798	ATG	TGA	266
Maturases	matR	1926	ATG	TAA	642
Transport membrance protein	mttB(2)	342	ATG	TGA	114
NADH dehydrogenase	nad1****	978	ACG(ATG)	TAA	326
	nad2(2)****	1467	ATG	TAA	489
	nad3	357	ATG	TAA	119
	nad4***	1488	ATG	TGA	496
	nad4L	303	ATG	TAA	101
	nad5(2)****	2013	ATG	TAA	671
	nad6	618	ATG	TAA	206
	nad7****	1185	ATG	TAG	395
	nad9	573	ATG	TAA	191
Ribosomal proteins (LSU)	rpl10	489	ATG	TAA	163
	rpl16	249	ATG	TAA	83
	rpl2	1014	ATG	TAA	338
	rpl5	564	ATG	TAA	188
Ribosomal proteins (SSU)	rps1	624	ATG	TAA	208
	rps12*	378	ATG	TGA	126
	rps14	303	ATG	TAG	101
	rps19	285	ATG	TAA	95
	rps3	1710	ATG	TAA	570
	rps4	1047	ATG	TAA	349
	rps7	447	ATG	TAA	149
Succinate dehydrogenase	sdh4	387	ATG	CGA(TGA)	129
Ribosomal RNAs	rrn18	1929			
	rrn26	3403			
	rrn5	121			
Transfer RNAs	trnA-TGC*	65			
	trnC-GCA(2)	71/73			
	trnD-GTC	74			
	trnE-TTC	72			
	trnF-GAA	74			
	trnG-GCC	72			
	trnH-GTG	74			
	trnI-GAT*	72			
	trnI-TAT*	76			
	trnK-TTT	73			
	trnM-CAT(3)	74/73/74			
	trnN-GTT	72			
	trnP-TGG(3)	74/75/75			
	trnQ-TTG	72			
	trnS-GCT	88			
	trnS-TGA(2)	93/87			
	trnV-GAC	72			
	trnW-CCA	74			
	trnY-GTA	83			

Note: Numbers after gene names are the number of copies. The superscripts * represent the number of contained introns

### Condon usage and RSCU analysis

The ATG was the most frequent start codon for protein-coding genes, while the *nad1* gene was an exception, having the initiating codon ACG (ATG). The stop codons TAA, TAG, TGA, and CGA (TGA) were identified (Table 1). These results indicated that the C to U RNA editing phenomenon was found in the start or stop codons.

**Fig. 2:**
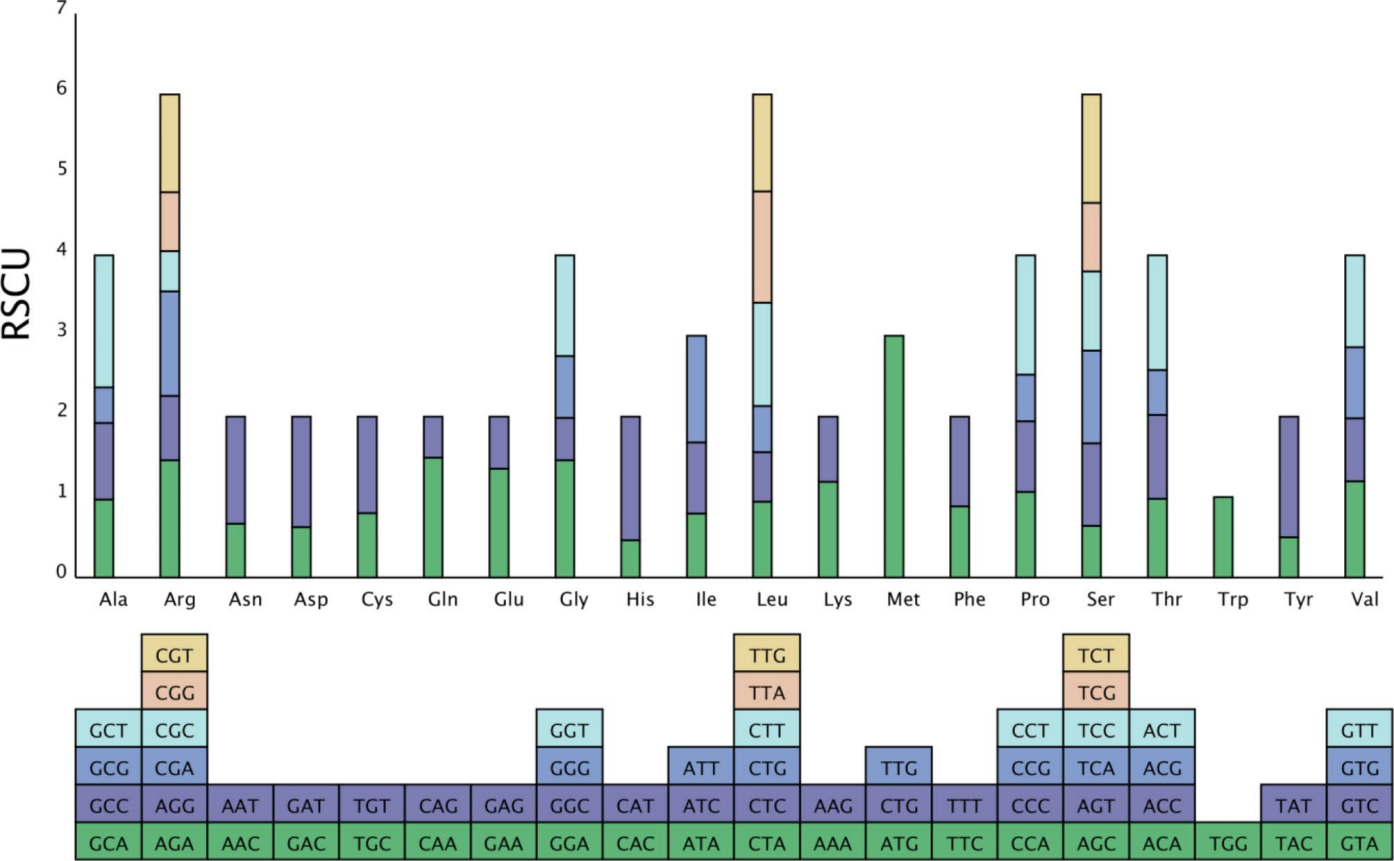
Relative synonymous codon usage (RSCU) in the *P. granatum* mitochondrial genome. The different amino acids are shown on the x-axis. RSCU values are the number of times a particular codon is observed relative to the number of times that codon would be expected for a uniform synonymous codon usage. The bottom square represents all the codons that code for each amino acid, and the height of the top column represents the sum of all the codon RSCU values

The relative synonymous codon usage (RSCU) analysis of *P. granatum* mitochondrial genome was shown in Fig. 2. It contained 10, 445 codons excluding termination codons in protein-coding genes regions. The most frequent codons used were UUU (Phe), AUU (Ile) and GAA (Glu) and were used>300 times, while CUG (Met), UUG (Met) and UAG (Ter) were rarely found (Table S2). We found that most RSCU values of the codons ending with A or T were higher than 1.0, while most of those ending with C or G had RSCU values of less than 1. Codon usage was generally strongly biased toward A or T (U) at the third codon position in the *P. granatum* mitogenome. Similar results had been found in the mitogenome of other plants [17, 27].

### Repeat sequence analysis

Dispersed repeats are repeats that are distributed at different locations in the genome. A total of 188 pairs of repeats with a length greater than or equal to 30 bp were found, including 97 pairs of reverse complementary repeats, 91 pairs of forward repeats. The longest reverse complementary repeat was 355 bp, while the longest forward repeat was 24,984 bp (Table S3).

**Fig. 3:**
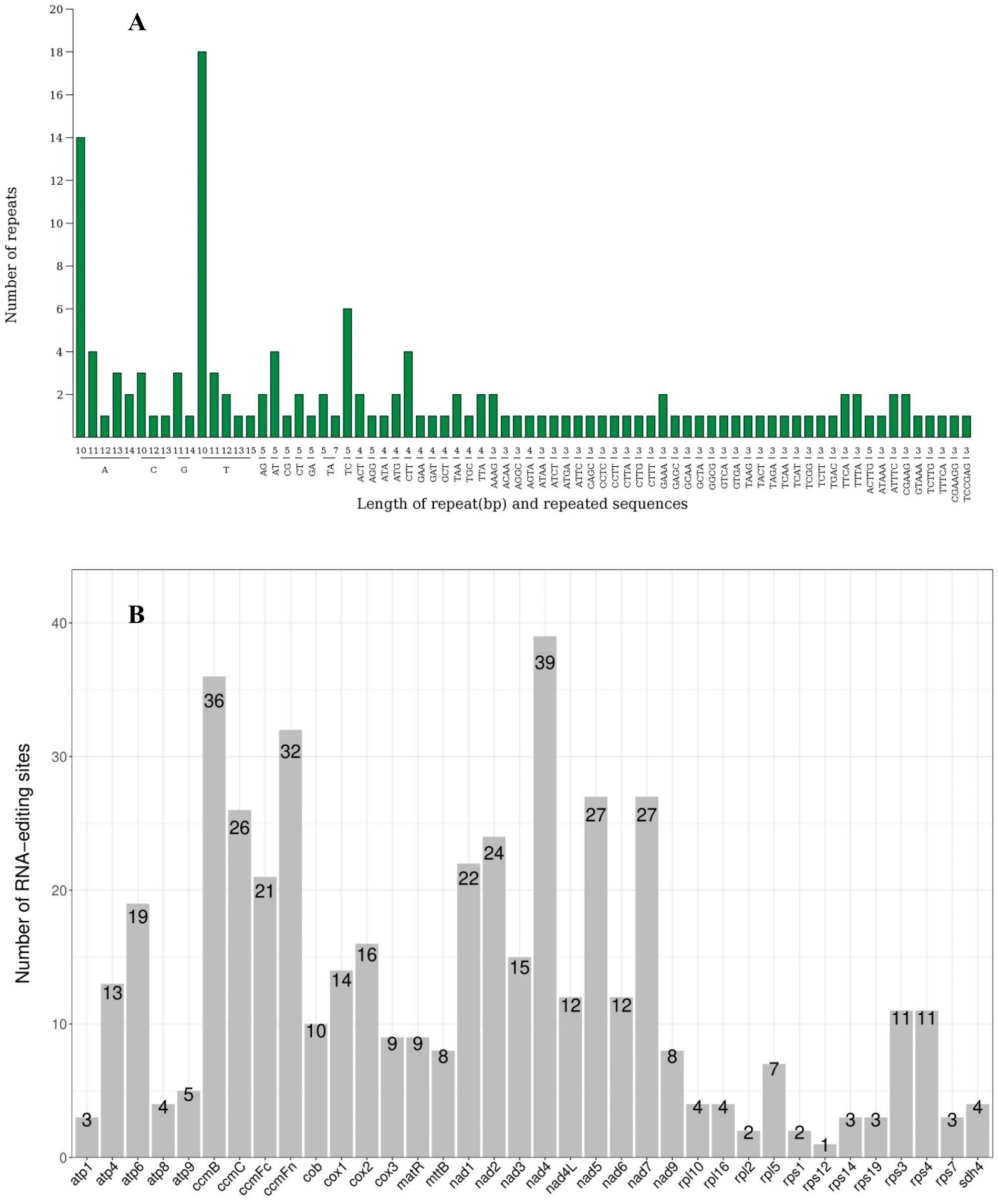
The number of SSR motifs and RNA editing sites predicted in *P. granatum* mitogenome. (**A**) The number of identified SSR motifs. **(B)** The number of RNA editing sites identified in each PCGs

The simple repeated sequences (SSRs) with motifs of one to six bases are abundant in higher plants. In the study, a total of 141SSRs were detected in the *P. granatum* mitogenome, including 58 (41.13%) mono-, 19 (13.48%) di-, 18 (12.77%) tri-, 35(24.82%) tetra-, 9 (6.38%) penta-, and 2 (1.42%) hexanucleotide repeats (Fig. 3A). Among them, monomeric and dimeric SSRs accounted for 54.61% of the total SSRs. Mononucleotide repeats of A/T (34.75%) were more prevalent than the other repeat types, and dinucleotide repeats of AG/CT (7.80%) were the second most numerous.

### Prediction of RNA editing sites

RNA editing events were identified for 36 unique PCGs based on online website predictions. There were 466 potential RNA editing sites distributed among all PCGs, and all of which were C-to-U base editing (Fig. 3B and Table S4). The *nad4* gene was the most edited and possessed 39 potential RNA editing sites of the mitochondrial genes. This was followed by the *ccmB* gene with 36 RNA editing events. The edited number of *rps1* gene was the lowest and had only one potential RNA editing events among all mitochondrial genes.

The total number of hydrophilic-hydrophobic type induced by RNA editing was 224 sites, which had the highest proportion at 48.07% (Table 2). The hydrophobic-hydrophobic and hydrophilic-hydrophilic type was 31.33% (146 sites) and 12.23% (57 sites), respectively. The hydrophilic-stop number was the lowest and the proportion was 1.07% (5 sites). Among them, there were 10 site conversions of CCT to TTT and 8 conversions of CCC to TTC.

**Table 2:** Prediction of RNA editing sites in *P. granatum* mitogenome

Type	RNA-editing	Number	Percentage
hydrophilic-hydrophilic	CAC (H) = > TAC (Y)	6	
	CAT (H) = > TAT (Y)	16	
	CGC (R) = > TGC (C)	6	
	CGT (R) = > TGT (C)	29	
	total	57	12.23%
hydrophilic-hydrophobic	ACA (T) = > ATA (I)	5	
	ACC (T) = > ATC (I)	1	
	ACG (T) = > ATG (M)	5	
	ACT (T) = > ATT (I)	4	
	CGG (R) = > TGG (W)	32	
	TCA (S) = > TTA (L)	71	
	TCC (S) = > TTC (F)	30	
	TCG (S) = > TTG (L)	38	
	TCT (S) = > TTT (F)	38	
	total	224	48.07%
hydrophilic-stop	CAA (Q) = > TAA (X)	3	
	CGA (R) = > TGA (X)	2	
	total	5	1.07%
hydrophobic-hydrophilic	CCA (P) = > TCA (S)	6	
	CCC (P) = > TCC (S)	8	
	CCG (P) = > TCG (S)	3	
	CCT (P) = > TCT (S)	17	
	total	34	7.30%
hydrophobic-hydrophobic	CCA (P) = > CTA (L)	36	
	CCC (P) = > CTC (L)	12	
	CCC (P) = > TTC (F)	8	
	CCG (P) = > CTG (L)	29	
	CCT (P) = > CTT (L)	23	
	CCT (P) = > TTT (F)	10	
	CTC (L) = > TTC (F)	8	
	CTT (L) = > TTT (F)	12	
	GCA (A) = > GTA (V)	1	
	GCG (A) = > GTG (V)	5	
	GCT (A) = > GTT (V)	2	
	total	146	31.33%
	All	466	100%

### Nucleotide diversity and comparative analysis of mitochondrial structure

**Fig. 4:**
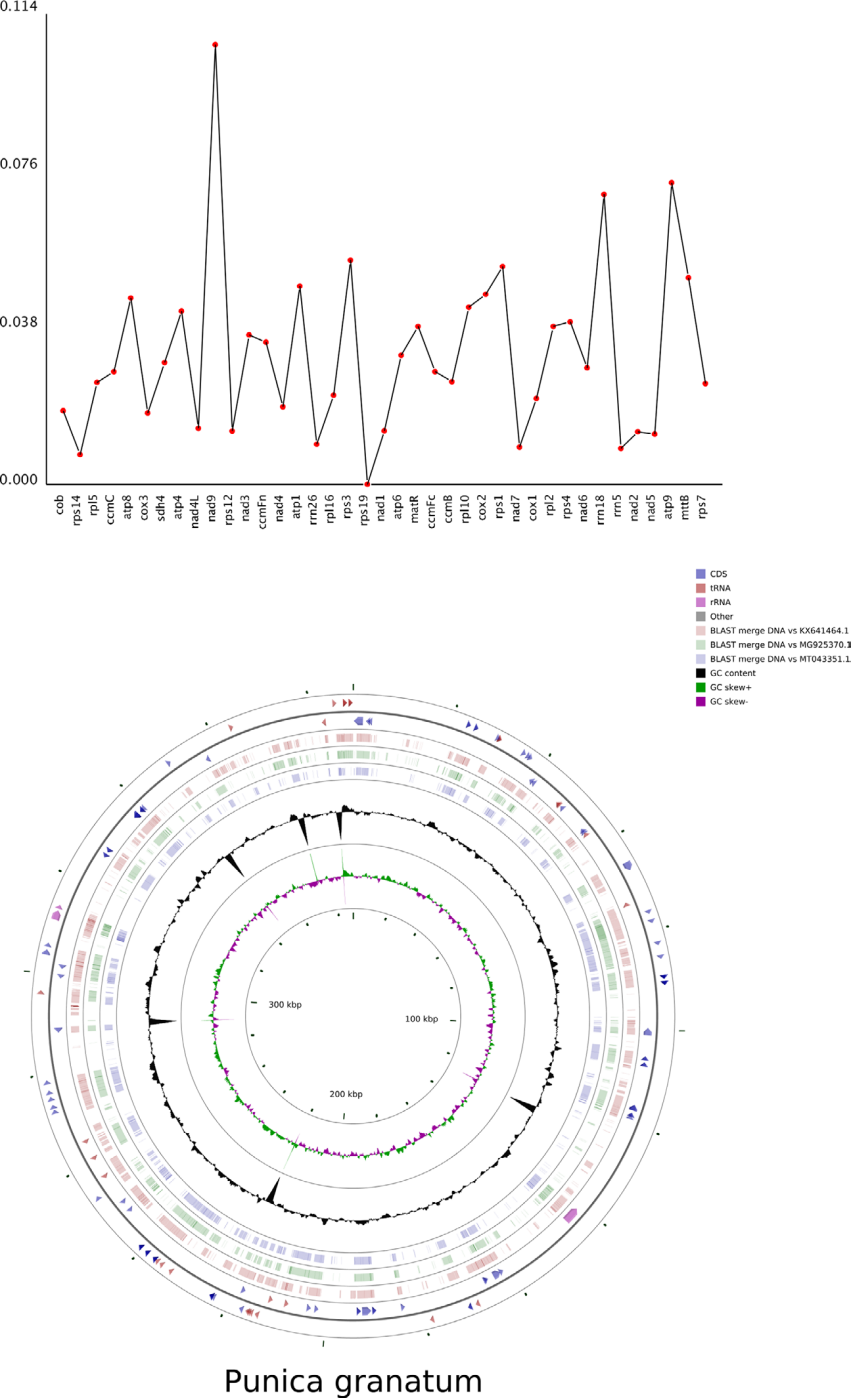
Nucleotide diversity and comparative analysis of *P. granatum* mitochondrial structure. (**A**) The gene nucleotide variability (pi) values. **(B)** Comparative analysis of mitochondrial structure of *P. granatum* and proximal species. The outer two circles describe the length and direction of the genome. The circles inside represent similar results compared to other reference genomes. The black circles represent the GC content

The nucleotide diversity (pi) values of 38 regions were calculated and ranged from 0 to 0.10582, with an average of 0.03132 (Fig. 4A and Table S5). The pi value of *gene10.nad9* region was highest in these regions, which was 0.07259 and 0.0698 in *gene37.atp9* and *gene33.rrn18* regions, respectively. The lower pi values suggested that the mitogenome sequences of *P. granatum* were highly conserved.

The mitogenome structures of *P. granatum* and its proximal species belonging to the Myrtales were comparatively analyzed using CGVIEW software (Fig. 4B). There were high similarities in the mitochondrial structure of *P. granatum* and *Eucalyptus grandis* (NCBI Number: MG925370.1), *Lagerstroemia indica* (NCBI Number: KX641464.1) and *Medinilla magnifica* (NCBI Number: MT043351.1).

### Phylogenetic and collinearity analysis

**Fig. 5:**
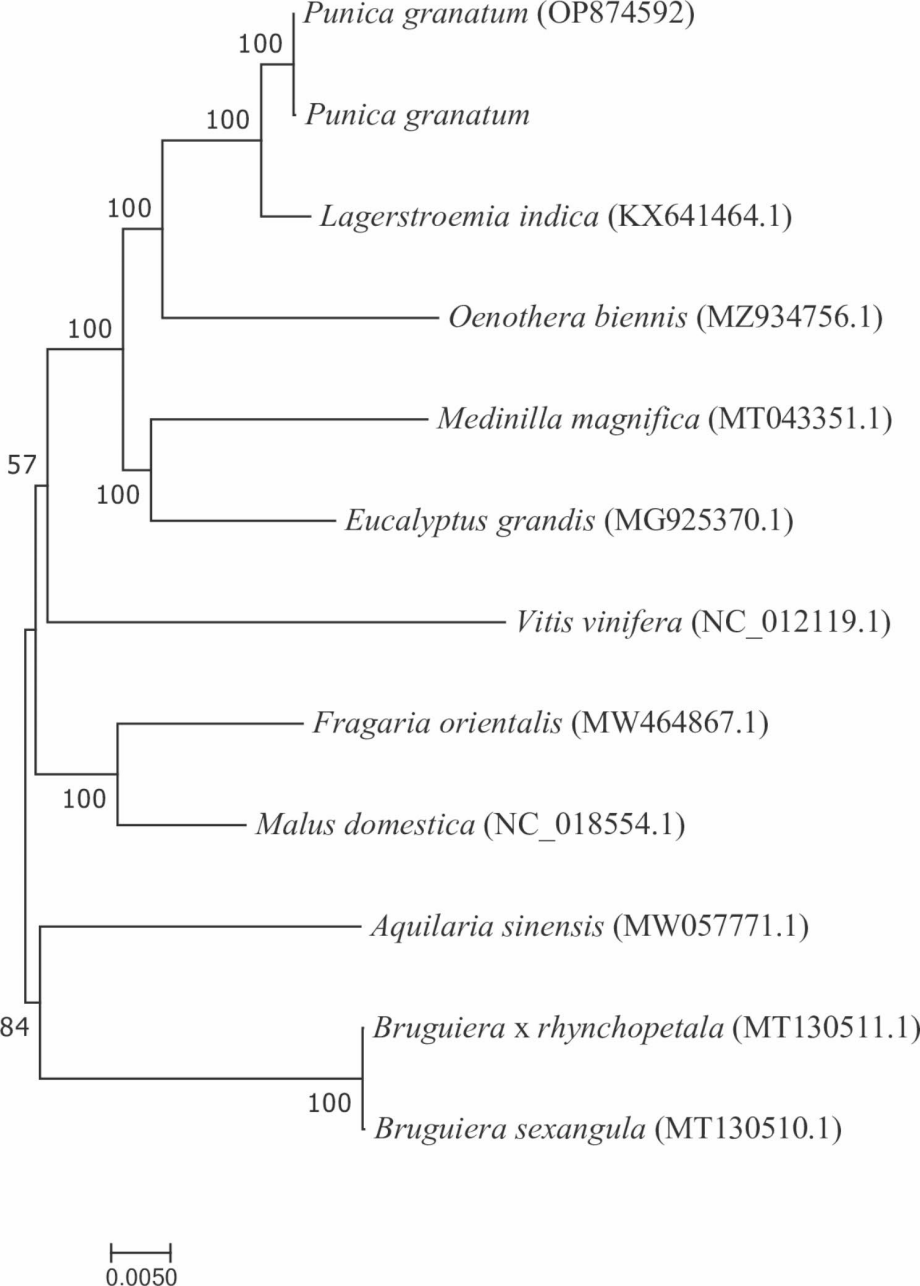
The evolutionary relationship between *P. granatum* and homologous species

The mitochondrial genome of proximate species of pomegranate is rarely reported. To further explore the evolutionary relationships of *P. granatum*, the phylogenetic tree was constructed by maximum likelihood method (Fig. 5). Phylogenetic analysis was performed on 30 conserved mitochondrial PCGs (*atp1*, *atp4*, *atp6*, *atp8*, *atp9*, *ccmC*, *ccmFc*, *ccmFn*, *cob*, *cox1*, *cox2*, *cox3*, *matR*, *nad1*, *nad2*, *nad3*, *nad4*, *nad4L*, *nad5*, *nad6*, *nad7*, *nad9*, *rpl5*, *rps1*, *rps3*, *rps4*, *rps12*, *rps14* and *sdh4*) from11 species. The mitochondrial genomes of *Malus domestica*, *Vitis vinifera* and *Fragaria orientalis* were set as outgroups. The phylogenetic tree strongly supports (100% bootstrap support) the close phylogenetic relationship between *P. granatum* ‘Taishanhong’ and *P. granatum* ‘Qingpitian’ and *Lagerstroemia indica*. The result indicated that the evolutionary relationship of mitochondrial genome among different pomegranate varieties is relatively close.

**Fig. 6:**
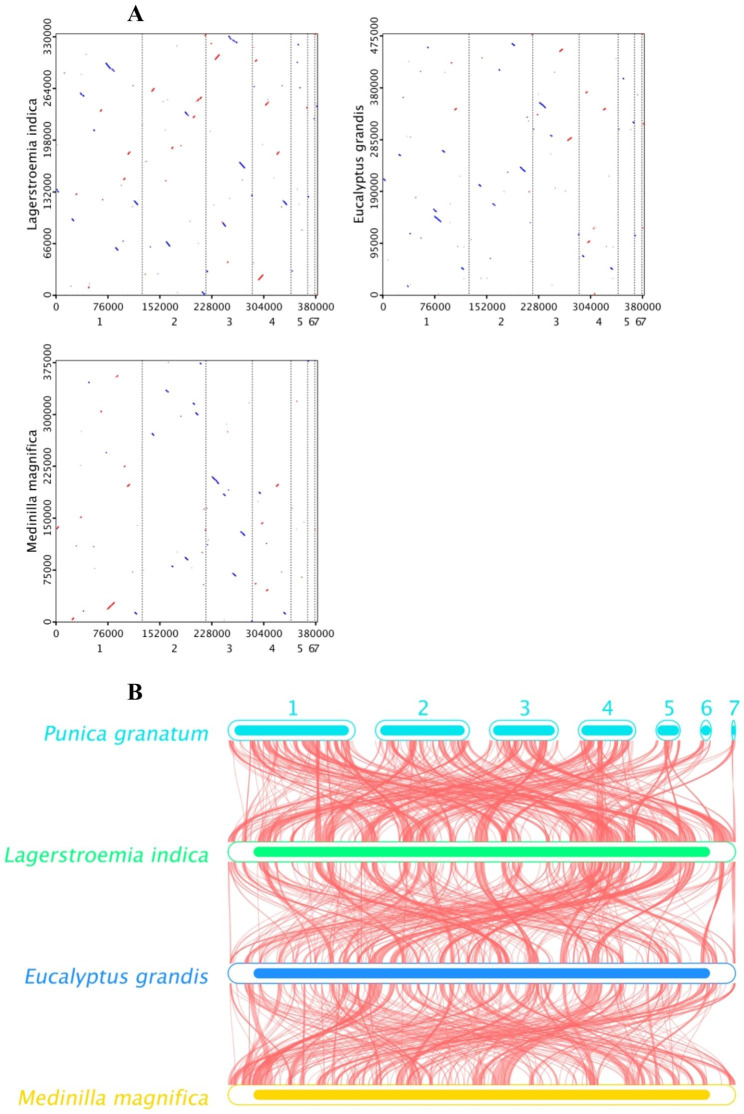
Collinear analysis of mitochondrial sequence with two methods. (**A**) The horizontal coordinate in each box indicates the assembled sequence, and the vertical coordinate indicates the other sequences. The red line in the box indicates the forward alignment, and the blue line indicates the reverse complementary alignment. **(B)** The boxes in each row represent a genome, and the lines in the middle represent homologous regions

The collinearity analysis of *Lagerstroemia indica*, *Eucalyptus grandis, Medinilla magnifica* and *P. granatum* were performed with two methods (Fig. 6). The mitogenome of *P. granatum* and *Lagerstroemia indica* had more collinear forward alignment and reverse complementary alignment sequences, which was followed by *P. granatum* and *Eucalyptus grandis* (Fig. 6A). There were many homologous collinear blocks between the P. granatum mitochondrial genome and other three proximate species (Fig. 6B). Some gaps illustrate that these sequences are unique to the species and have no homology with the rest of the species. The results suggest that the *P. granatum* mitochondrial genome has undergone a lot of genomic rearrangement with close species.

### Intracellular gene transfer of *P. granatum* organelle genomes

**Fig. 7:**
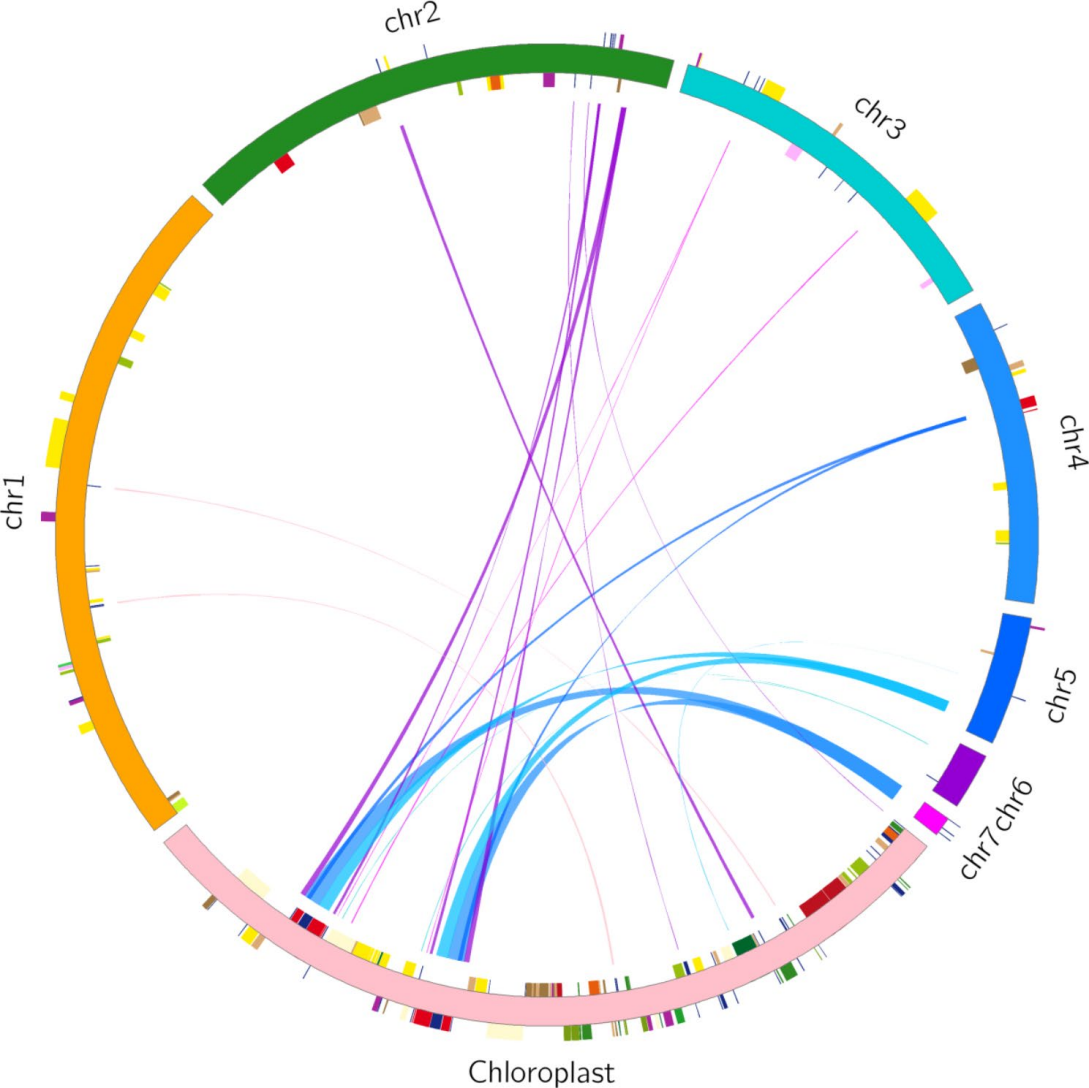
Homologous fragments of chloroplast and mitochondrial sequence in *P. granatum.* Chloroplast is chloroplast sequence and chr is mitochondrial sequence. Genes from the same complex are labeled with the same color, with homologous sequences represented at the center line junctions

The *P. granatum* mitogenome sequence was approximately 2.41 times longer than its cp. genome (NCBI Number: MK603511, 1158,638 bp). According to sequence similarity analysis, there were 22 homologous fragments between the mitogenome and chloroplast genome, with a total length of 19,889 bp, accounting for 5.20% of the total mitogenome (Fig. 7 and Table S6). By annotating these homologous sequences, 11 complete genes were also found on 22 homologous fragments, including nine tRNA genes (*trnD-GUC*, *trnH-GUG*, *trnI-GAU*, *trnM-CAU*, *trnP-UGG*, *trnS-UGA*, *trnV-GAC*, *trnW-CCA*, *trnN-GUU*) and two rRNA genes (*rrn5* and *rrn16*) (Table S6). There were 35 gene sequences transferred from the chloroplast to the mitochondria in *P. granatum* (Table S7). The length of *atpA_len507* gene was the longest with a length of 471 bp. The *ndhA_len363* gene had the shortest length (35 bp).

## Discussion

### Structure and characteristic of *P. granatum* mitogenome

The genome structure of plant mitochondrial genomes has always been mapped as a single master circle and a collection of subgenomic circles because of the large, direct, repeat recombination [28, 29]. For example, the mitochondrial genomes of bitter gourd [11] and *Vitex rotundifolia* [30] were mapped as a molecular circle. The mitochondrial genome of *Chaenomeles speciosa* was multi-chromosomes with two circular contigs [31] and *Paphiopedilum micranthum* with 26 circular contigs [32], while the *Q. acutissima* mitogenome was a branched structure of two circular molecules and one linear molecule [33]. In the present study, the *P. granatum* ‘Taishanhong’ mitogenome was represented by seven circular contigs structure (Fig. 1) with a total length of 382,774 bp. There was significant difference in the structure (a single linear molecule) and size (404,807 bp) of *P. granatum* ‘Qingpi Sweet’ mitogenome [26]. The difference in genome size may be related to the different mitochondrial structure of the two varieties. This suggested that the mitochondrial genomes of pomegranate were species specific and possessed many unique features, which is consistent with research on other species [17, 21]. The result provided new insights into the function and structure of the mitochondrial genomes in pomegranate species.

GC content is an important factor for assessing species. The total GC content of *P. granatum* mitogenome was 45.91%, which was highest in the rRNA genes. The result was comparable to that of *Phaseolus vulgaris* (45.11%) [16], Bitter Gourd (45.60%) [11], and *Bupleurum chinense* (45.68) [34], which was higher than that in *Mangifera sylvatica* (44.66%) [21]. The mitogenome of *P. granatum* ‘Taishanhong’ encoded 74 genes, including 46 protein-coding genes, 25 tRNA genes, and three rRNA genes, which was higher than that in *P. granatum* ‘Qingpi Sweet’(60 genes) [26].

The intron contents of the plant mitochondrial genes are conserved in each lineage but are variable among different lineages [35]. In the present study, the trans-spliced genes *nad1*, *nad2*, *nad5*, n*ad7* contained four introns, and *nad4* gene had three introns. These trans-spliced introns were postulated to have evolved from one common ancestor by fragmentation of a cis-spliced arrangement [36].

### Identification of condon usage and repeat sequences

Most PCGs were the typical ATG start codon, while n*ad1* gene uses the ACG as initiation codons in the *P. granatum* mitogenome. The phenomenon also has been found in other studies, which were considered to be altered by RNA editing modification [37, 38].

Codon usage preference is usually considered to be the result of the tendency to develop relative equilibrium within cells during species evolution, and codons with a RSCU greater than 1 are considered to be used by amino acid preference [17, 39–41]. The result indicated that Phe, Ile and Glu were the most common amino acids, and Met and Ter were much less common in the *P. granatum* mitogenome. The most prevalent amino acids were Ala, Tyr, and His in *Camellia sinensis* [42], while Ala, Gln, and His were the most common amino acids in *Clematis acerifolia* [43].

Repeated sequences are widely present in the mitochondrial genome, which are generally vital for intermolecular recombination, structural variations and extreme mitogenome sizes [44–46]. In this study, a total of 188 pairs of repeats were observed, including 97 pairs of reverse complementary repeats and 91pairs of forward repeats. These repetitive sequences play a crucial role in the assembly of the *P. granatum* mitogenome [47]. Simple sequence repeats are a special kind of presence among tandem repeats, which can better reflect species’ genetic structure and genetic diversity changes [48, 49]. There was a total of 141 SSRs were detected and mononucleotide repeats of A/T were the most frequent, which was consistent with other research findings in *Momordica charantia* [11] and *Calla Lilies* [28]. It provided a large number of reference markers for the evaluation of pomegranate germplasm diversity and species identification.

### RNA editing analysis

RNA editing is a very common phenomenon in the mitochondrial genomes of higher plants. Almost all of the transcription products of mitochondrial PCGs are subjected to varying degrees of RNA editing but rarely occur in rRNA, tRNA, and introns [50–52]. RNA editing can affect the composition of the final mitochondrial proteome, which can create start and stop codons in the mitochondrial mRNA molecules [53, 54]. Previous researches had uncovered approximately 421RNA editing sites within 26 genes in *Acer truncatum* [17] and 486 RNA editing sites within 31 genes in *Phaseolus vulgaris* [16]. There were 466 potential RNA editing sites distributed among 36 PCGs, and all were C-to-U base editing in *P. granatum* mitogenome. The result was similar to the study in *Salvia officinalis* [55], *Leucaena trichandra* [56] and *Pereskia aculeata* [57]. The *nad4* gene possessed 39 potential RNA editing sites and was the most edited of pomegranate mitogenome, which was supported by other studies [14, 41].

### Phylogenetic relationships and synteny analysis

Changes in the size and structure of plant mitochondrial genomes are evident, but functional genes remain conserved [58]. The nucleotide diversity (pi) reveals the variation of nucleotide sequence in different species (Fig. 4A). Our data showed that the pi value of *nad9* gene region was highest in these regions. The lower pi values suggested that the mitogenome sequences of *P. granatum* were highly conserved.

There were high similarities in the mitochondrial structure of *P. granatum* and *Eucalyptus grandis*, *Lagerstroemia indica* and *Medinilla magnifica* (Fig. 4B). The phylogenetic tree strongly supports the close phylogenetic relationship between *P. granatum* and *Lagerstroemia indica*. It was further confirmed from the molecular level that the pomegranate belonged to the Lythraceae family [59]. The co-linearity between *P. granatum* and *Lagerstroemia indica* was also high with large homozygous co-linear blocks, suggesting that the two species have undergone extensive rearrangement phenomena in the long evolutionary process [60]. The result provided an effective way to understand species evolution of pomegranate, and the specific mechanism and process need to be further studied.

### Intracellular gene transfer of *P. granatum* organelle genomes

Gene transfer from chloroplasts to mitochondrial genomes is common during long-term plant evolution. The discovery of intracellular gene transfers can contribute to the accurate assembly of chloroplast and mitochondrial genomes and to the understanding of organelle genome evolution [61, 62]. We found 22 homologous fragments between the pomegranate mitogenome and chloroplast genome, with a total length of 19,889 bp, accounting for 5.20% of the total mitogenome. There were 35 gene sequence of chloroplast transfer to mitochondria in *P. granatum*, while the length of atpA_len507 gene was the longest with a length of 471 bp. Previous studies have found that tRNA genes occasionally migrated from the chloroplast to mitochondria, such as *trnD-GUC*, *trnN-GUU*, and *trnH-GUG* in *Acer truncatum* [17], *trnV-GAC*, *trnP-TGG*, *trnI-GAU*, and *trnA-UGC* in *Camellia sinensis* [42], etc. The nine complete tRNA genes of homologous fragments were identified from the chloroplast of *P. granatum*, which suggested that they may play a role in normal functions [63]. Therefore, larger-scale sampling is required better to understand the evolution of phylogenetic analyses and molecular markers of pomegranate. These studies will provide a substantial foundation for understanding pomegranate mitogenome evolution and facilitate mitochondrial-based breeding.

## Conclusions

In this study, we assembled and annotated the complete mitogenome of *P. granatum* cultivar ‘Taishanhong’. The genome structure was multi-chromosomes with seven circular chromosomes, and total length was 382,774 bp and GC content was 45.91%. In addition, the codon usage, sequence repeats, RNA editing and chloroplast to mitochondrion DNA transformation were also analyzed. There were high similarities in the mitochondrial structure between *P. granatum* and its proximal species. Evolutionary and collinear analysis showed that *P. granatum* was closely related to *P. granatum ‘*Qingpitian*’* and *Lagerstroemia indica*. This study provided information on the genetic characteristics, phylogenetic relationships, species identification and biological evolution of *P. granatum*.

## Methods and materials

### Plant materials

The tissue culture seedlings of *P. granatum* cultivar ‘Taishanhong’ were planted in the tissue culture room at Shandong Institute of Pomology (Taian, Shandong, China). Plants were kept in the dark for 14 d to obtain etiolated seedlings. Then, the fresh leaves were obtained and cleaned with DEPC water and kept in a freezer at -80 °C.

### DNA extraction and sequencing

The second and third generation sequencing strategy was adopted to complete the assembly of mitochondrial genome [55]. The total genomic DNA was extracted by a DNA plant extraction kit (Tiangen, Beijing, China). After the genomic DNA of the sample was qualified, the DNA was fragmented using ultrasound. DNA fragments were purified, terminal repair, A was added to the 3 ‘end, and sequencing splices were connected. The fragment size was selected by agarose gel electrophoresis and PCR amplification was performed to form sequencing library. The constructed libraries were inspected first, and second qualified libraries were sequenced using Illumina Novaseq6000 platform by Nanjing Genepioneer Biotechnologies (Nanjing, China). The sequencing was performed as paired-end and read length was 150 bp. The Fastp (version 0.20.0, https://github.com/OpenGene/fastp) was used to filter the raw data, removing primer sequences and sequencing junctions, discarding reads with quality scores below Q5, and removing N values greater than 5. Finally, we obtained a total of 21, 083,687clean reads and the GC content was 40.21%. The Q20 and Q30 were respectively 96.39% and 90.68%.

After the samples passed the quality inspection, the Third generation sequencing experiment was performed. The genomic DNA is randomly interrupted, and large fragments of DNA are enriched and purified by magnetic beads. Then, the large fragments are cut and recovered, and the damage of fragmented DNA is repaired. After purification, the end repair was performed on both ends of the DNA fragment and A was added. The joint in the SQK-LSK109 connection kit was used for the connection reaction. Finally, the constructed DNA library was quantitatively detected. After the DNA library with a certain concentration and volume was added into the Flow cell, and was transferred to the Oxford Nanopore PromethION sequencer for real-time single molecule sequencing. The three generations of sequencing data were filtered using filtlong v0.2.1 software, and counted using Perl scripts.

### Assembly and annotation of the mitochondrial genome

The minimap2 v2.1 software was used to compare the reference gene sequence (plant mitochondrial core gene, the company’s own core genetics website: https://github.com/xul962464/plant_mt_ref_gene) with the original third-generation data [64]. The sequences with alignment length greater than 50 bp were selected as candidate sequences, and the sequences with more alignment genes and higher alignment quality were selected as seed sequences. The sequences with overlap greater than 1 kb and similarity greater than 70% were screened and obtained all third-generation sequencing data of mitochondrial genome. The canu software [4] was used to correct the obtained third-generation data and bowtie2 (v2.3.5.1) was used to compare the second-generation data to the corrected sequence [65]. The Unicycler (v0.4.8) default parameter comparison was used to concatenate the second-generation data and the third-generation data after correction. The corrected third-generation sequencing data was compared to the contig obtained in the second step of Unicycler using minimap2, and the branch direction was manually determined to obtain the final assembly result.

The encoded proteins and rRNAs were compared to published plant mitochondrial sequences using BLAST, and further manual adjustments were made based on closely related species. The tRNA was annotated using tRNAscanSE (http://lowelab.ucsc.edu/tRNAscan-SE/) software [66]. ORFs were annotated using Open Reading Frame Finder (http://www.ncbi.nlm.nih.gov/gorf/gorf.html). The minimum length was set as 102 bp, and redundant sequences and sequences with overlap with known genes were excluded. Sequences with length greater than 300 were compared with the nr library and annotated. The mitochondrial genome map was constructed using OGDRAW (https://chlorobox.mpimp-golm.mpg.de/OGDraw.html). RNA editing sites were predicted using the Plant Predictive RNA Editor (PREP) suite (http://prep.unl.edu/). Because the site has to stop the service, so we according to the algorithm and database to rewrite a tool PmtREP (http://112.86.217.82:9919/#/tool/alltool/detail/336), which is used to plant mitochondrial genome RNA editing sites prediction.

### Analysis of RSCU and repeat sequences

Relative Synonymous Codon Usage (RSCU) is the ratio of the actual frequency of the use codon/theoretical frequency of the use codon. The RSCU of the mitochondrial genome of *P. granatum* was analyzed using a self-encoded Perl script, to screen for a unique CDS and determine the number of codons per gene. The blastn (v2.10.1) software was used to identify the repeat sequence of the genome itself. The -word_size was set to 7 and evalue was set to 1e-5, and the result was obtained after removing the redundancy.

### Phylogenetic tree construction

The CDS was used to make the maximum likelihood evolutionary tree. MAFFT software (v7.427) was used for multi-sequence alignment of inter-species sequences. The matched sequences were connected from head to tail, and trimAl (v1.4.rev15) was used to trim. The jmodeltest-2.1.10 software was used to predict the model after trimming, and the model was determined as GTR type. The maximum likelihood evolutionary tree was constructed using RAxML v8.2.10 (https://cme.h-its.org/exelixis/software.html) software. The GTRGAMMA model and bootstrap = 1000 were selected as the parameter.

### Genomic comparison analysis with related species

The Mafft software was used for global alignment of homologous gene sequences of different species, and dnasp5 was used to calculate the pi value of each gene. The comparative analysis of mitochondrial genome structure between *P. granatum* and proximal species was carried out using the CGVIEW (http://stothard.afns.ualberta.ca/cgview_server/) software. The nucmer (4.0.0beta2) software with maxmatch parameter was used for genome alignment between other sequences and assembly sequences, and Dot-plot was generated. The blastn (2.10.1+) software was used to compare the assembled species and the selected species in turn, and Multiple Synteny plots were drawn. The parameters were set as follows: -word_size was 7 and E-value was 1e-5. The blast software was used to find homologous sequences between chloroplasts and mitochondria genome, and the similarity was set to 70% with an E-value of 1E-5. The homologous fragments were visualized using circos v0.69-5 software.

### Electronic supplementary material

Below is the link to the electronic supplementary material.

**Fig. S1**. Branched conformation of *P. granatum* mitogenome. **Fig. S2**. Data analysis depth chart of *P. granatum* mitogenome. **Supplementary Tables: Table S1**. General features of the *P. granatum* mitogenome. **Table S2**. Codon usage of *P. granatum* mitogenome. **Table S3**. Repeated sequence analysis of *P. granatum* mitogenome. **Table S4**. The RNA editing events prediction in *P. granatum* mitogenome. **Table S5**. The nucleotide variability of *P. granatum* mitogenome. **Table S6**. Comparison information of chloroplast and mitochondrial genome in *P. granatum*. **Table S7**. Gene sequence of chloroplast transfer to mitochondria in *P. granatum*.

## Data Availability

The raw sequencing data from the Illumina and Nanopore platforms generated during this study are available in GenBank. The associated BioProject and BioSample and SRA numbers are PRJNA971780 and SAMN35054861. SRA numbers of second and third generation data for the Illumina sequencing reads are SRR24519284 and SRR24554674, respectively. The mitogenome sequences were released into GenBank soon with the following accession numbers: OQ973289, OQ973290, OQ973291, OQ973292, OQ973293, OQ973294 and OQ973295. The plant samples are stored at the Shandong Institute of Pomology, Shandong, China (voucher numbers: SD-SL-026).
