# Supplementary material for: Multichromosomal mitochondrial genome of Punica granatum: comparative evolutionary analysis and gene transformation from chloroplast genomes
Source: BMC Plant Biol. 2023 Oct 25;23:512. doi: 10.1186/s12870-023-04538-8 (PMC10598957; doi:10.1186/s12870-023-04538-8)
Supplement: Supplementary file 1 — Fig. S1. Branched conformation of P. granatum mitogenome. Fig. S2. Data analysis depth chart of P. granatum mitogenome. Supplementary Tables: Table S1. General features of the P. granatum mitogenome. Table S2. Codon usage of P. granatum mitogenome. Table S3. Repeated sequence analysis of P. granatum mitogenome. Table S4. The RNA editing events prediction in P. granatum mitogenome. Table S5. The nucleotide variability of P. granatum mitogenome. Table S6. Comparison information of chloroplast and mitochondrial genome in P. granatum. Table S7. Gene sequence of chloroplast transfer to mitochondria in P. granatum. [file 12870_2023_4538_MOESM1_ESM.docx]

Supplementary Material


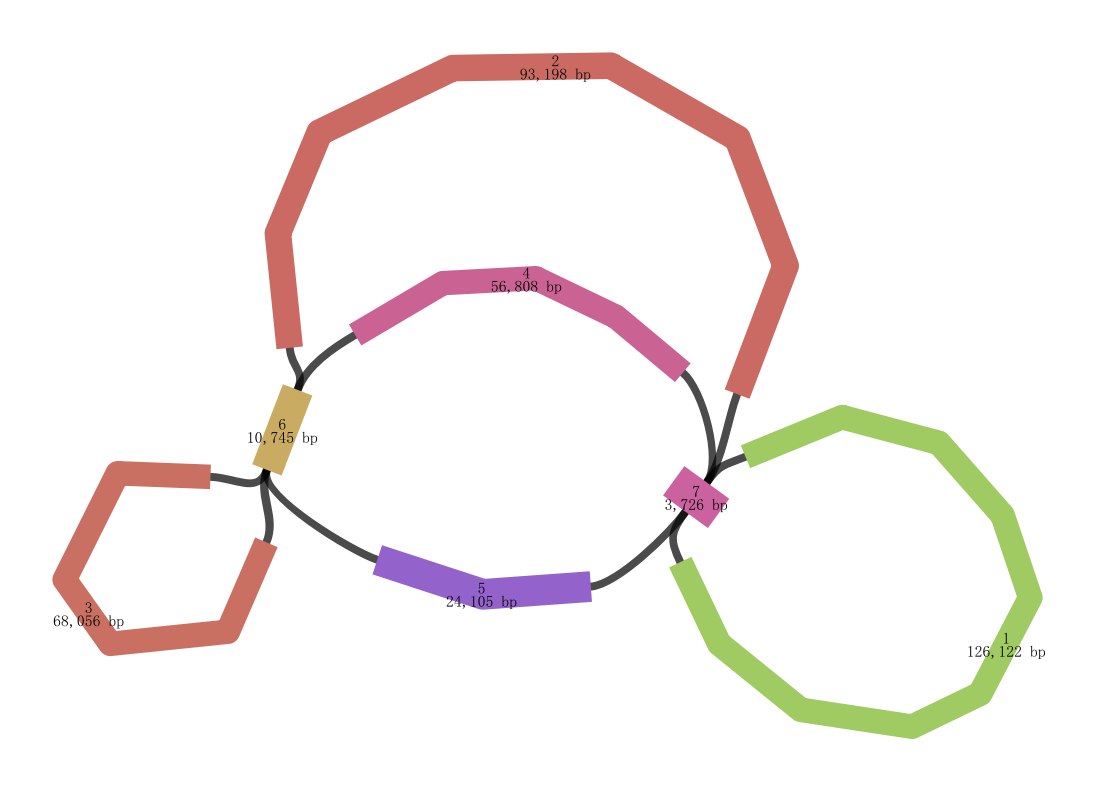


**Fig. S1.** Branched conformation of *P. granatum* mitogenome.


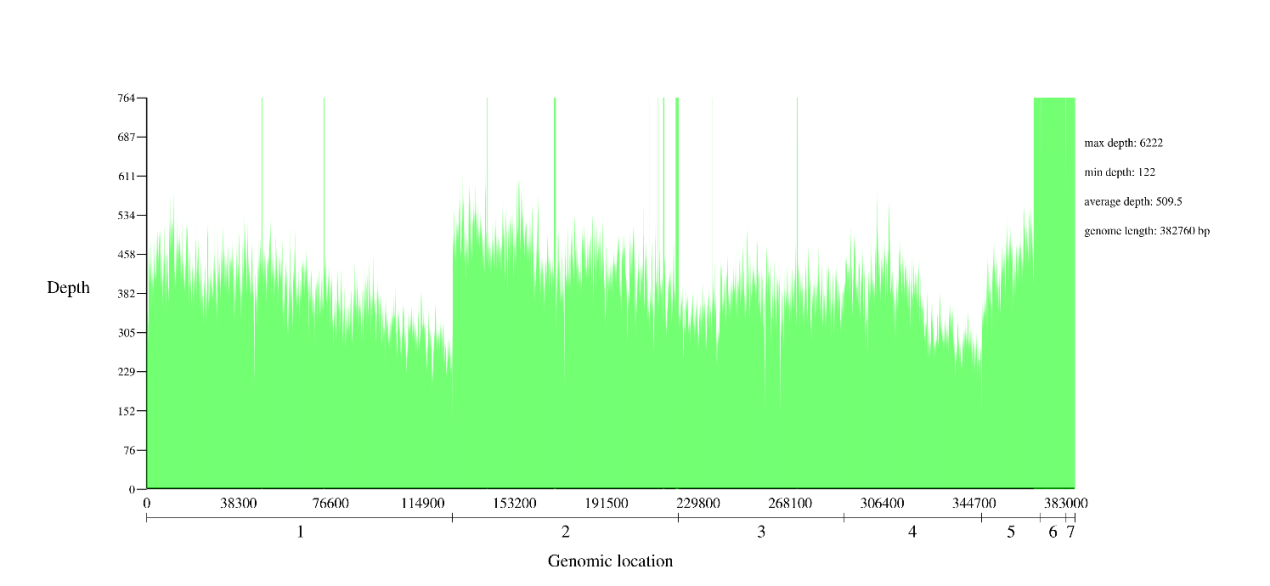


**Fig. S2.** Data analysis depth chart of *P. granatum* mitogenome.

**Supplementary Tables**

**Table S1.** **General features of the *P. granatum* mitogenome**

| ID | Type | Length | GC% | CDS_number | CDS_  length | CDS_GC(%) | tRNA_ number | tRNA_ length | tRNA_GC(%) | rRNA_ number | rRNA_ length | rRNA_GC(%) |
| --- | --- | --- | --- | --- | --- | --- | --- | --- | --- | --- | --- | --- |
| Punica_granatum_chr1 | linear | 126124 | 45.42 | 20 | 18840 | 42.06 | 5 | 372 | 51.08 | 0 | 0 | 0 |
| Punica_granatum_chr2 | linear | 93200 | 45.60 | 9 | 8361 | 44.74 | 8 | 624 | 50.48 | 1 | 3403 | 51.22 |
| Punica_granatum_chr3 | linear | 68058 | 46.74 | 9 | 10959 | 41.30 | 6 | 443 | 50.79 | 0 | 0 | 0 |
| Punica_granatum_chr4 | linear | 56810 | 46.10 | 6 | 6384 | 41.68 | 1 | 73 | 47.95 | 2 | 2050 | 53.66 |
| Punica_granatum_chr5 | linear | 24107 | 46.03 | 2 | 789 | 43.73 | 1 | 87 | 50.57 | 0 | 0 | 0 |
| Punica_granatum_chr6 | linear | 10747 | 45.48 | 0 | 0 | 0 | 1 | 74 | 54.05 | 0 | 0 | 0 |
| Punica_granatum_chr7 | linear | 3728 | 52.60 | 0 | 0 | 0 | 3 | 209 | 51.67 | 0 | 0 | 0 |
| Punica_granatum_Total | - | 382774 | 45.91 | 46 | 45333 | 42.35 | 25 | 1882 | 50.85 | 3 | 5453 | 52.14 |

**Table S2.** **Codon usage of *P. granatum* mitogenome**

| Amino Acid | Symbol | Codon | Number of uses | RSCU |
| --- | --- | --- | --- | --- |
| * | Ter | UAA | 24 | 2 |
| * | Ter | UAG | 3 | 0.25 |
| * | Ter | UGA | 9 | 0.75 |
| A | Ala | GCA | 159 | 0.9666 |
| A | Ala | GCC | 157 | 0.9544 |
| A | Ala | GCG | 72 | 0.4377 |
| A | Ala | GCU | 270 | 1.6413 |
| C | Cys | UGC | 60 | 0.8 |
| C | Cys | UGU | 90 | 1.2 |
| D | Asp | GAC | 108 | 0.6261 |
| D | Asp | GAU | 237 | 1.3739 |
| E | Glu | GAA | 302 | 1.3512 |
| E | Glu | GAG | 145 | 0.6488 |
| F | Phe | UUC | 295 | 0.8859 |
| F | Phe | UUU | 371 | 1.1141 |
| G | Gly | GGA | 263 | 1.4571 |
| G | Gly | GGC | 95 | 0.5263 |
| G | Gly | GGG | 138 | 0.7645 |
| G | Gly | GGU | 226 | 1.2521 |
| H | His | CAC | 55 | 0.4641 |
| H | His | CAU | 182 | 1.5359 |
| I | Ile | AUA | 211 | 0.7952 |
| I | Ile | AUC | 234 | 0.8819 |
| I | Ile | AUU | 351 | 1.3229 |
| K | Lys | AAA | 282 | 1.1874 |
| K | Lys | AAG | 193 | 0.8126 |
| L | Leu | CUA | 174 | 0.9405 |
| L | Leu | CUC | 114 | 0.6162 |
| L | Leu | CUG | 106 | 0.573 |
| L | Leu | CUU | 237 | 1.2811 |
| L | Leu | UUA | 256 | 1.3838 |
| L | Leu | UUG | 223 | 1.2054 |
| M | Met | AUG | 280 | 3 |
| M | Met | CUG | 0 | 0 |
| M | Met | UUG | 0 | 0 |
| N | Asn | AAC | 114 | 0.6686 |
| N | Asn | AAU | 227 | 1.3314 |
| P | Pro | CCA | 151 | 1.0653 |
| P | Pro | CCC | 124 | 0.8748 |
| P | Pro | CCG | 82 | 0.5785 |
| P | Pro | CCU | 210 | 1.4815 |
| Q | Gln | CAA | 218 | 1.4881 |
| Q | Gln | CAG | 75 | 0.5119 |
| R | Arg | AGA | 175 | 1.4563 |
| R | Arg | AGG | 96 | 0.7989 |
| R | Arg | CGA | 156 | 1.2982 |
| R | Arg | CGC | 60 | 0.4993 |
| R | Arg | CGG | 88 | 0.7323 |
| R | Arg | CGU | 146 | 1.215 |
| S | Ser | AGC | 102 | 0.6415 |
| S | Ser | AGU | 163 | 1.0252 |
| S | Ser | UCA | 183 | 1.1509 |
| S | Ser | UCC | 156 | 0.9811 |
| S | Ser | UCG | 136 | 0.8553 |
| S | Ser | UCU | 214 | 1.3459 |
| T | Thr | ACA | 128 | 0.979 |
| T | Thr | ACC | 136 | 1.0402 |
| T | Thr | ACG | 73 | 0.5583 |
| T | Thr | ACU | 186 | 1.4226 |
| V | Val | GUA | 193 | 1.1932 |
| V | Val | GUC | 127 | 0.7852 |
| V | Val | GUG | 143 | 0.8841 |
| V | Val | GUU | 184 | 1.1376 |
| W | Trp | UGG | 159 | 1 |
| Y | Tyr | UAC | 80 | 0.5031 |
| Y | Tyr | UAU | 238 | 1.4969 |

**Table S3.** **Repeated sequence analysis of *P. granatum* mitogenome**

| Chr1 | Chr2 | type | Alignment length | similarity | start1 | end1 | start2 | end2 | E value |
| --- | --- | --- | --- | --- | --- | --- | --- | --- | --- |
| 1 | 1 | P | 342 | 86.55 | 67323 | 67663 | 4407 | 4732 | 8.84E-99 |
| 1 | 1 | P | 355 | 89.915 | 4407 | 4732 | 67310 | 67663 | 8.84E-99 |
| 1 | 1 | P | 80 | 95 | 92518 | 92597 | 60478 | 60557 | 3.51E-28 |
| 1 | 1 | P | 77 | 96.104 | 60481 | 60557 | 92518 | 92594 | 3.51E-28 |
| 1 | 1 | F | 67 | 97.015 | 15259 | 15325 | 13243 | 13309 | 2.73E-24 |
| 1 | 1 | P | 81 | 91.358 | 22292 | 22370 | 4726 | 4802 | 4.57E-22 |
| 1 | 1 | P | 48 | 100 | 106279 | 106326 | 24909 | 24956 | 4.60E-17 |
| 1 | 1 | F | 39 | 100 | 827 | 865 | 719 | 757 | 4.64E-12 |
| 1 | 1 | P | 48 | 93.75 | 108154 | 108201 | 49005 | 49052 | 4.64E-12 |
| 1 | 1 | P | 58 | 87.931 | 101324 | 101380 | 34926 | 34983 | 2.16E-10 |
| 1 | 1 | F | 36 | 100 | 89246 | 89281 | 87536 | 87571 | 2.16E-10 |
| 1 | 1 | F | 75 | 82.667 | 899 | 972 | 758 | 832 | 7.76E-10 |
| 1 | 1 | P | 101 | 79.208 | 43738 | 43831 | 5160 | 5260 | 2.79E-09 |
| 1 | 1 | F | 37 | 97.297 | 52465 | 52501 | 17360 | 17396 | 2.79E-09 |
| 1 | 1 | P | 51 | 88.235 | 108177 | 108227 | 35326 | 35376 | 1.00E-08 |
| 1 | 1 | P | 62 | 83.871 | 52500 | 52561 | 15259 | 15320 | 3.61E-08 |
| 1 | 1 | P | 47 | 89.362 | 118276 | 118322 | 22528 | 22574 | 3.61E-08 |
| 1 | 1 | P | 30 | 100 | 119376 | 119405 | 21913 | 21942 | 4.67E-07 |
| 1 | 1 | P | 33 | 96.97 | 55653 | 55685 | 29246 | 29278 | 4.67E-07 |
| 1 | 1 | F | 33 | 96.67 | 81404 | 81436 | 48077 | 48109 | 4.67E-07 |
| 1 | 4 | F | 24984 | 100 | 101139 | 126122 | 31826 | 56808 | 0 |
| 1 | 4 | F | 104 | 94.231 | 80471 | 80574 | 10346 | 10449 | 3.46E-38 |
| 1 | 4 | F | 75 | 98.667 | 81315 | 81389 | 22557 | 22631 | 2.10E-30 |
| 1 | 4 | P | 84 | 90.476 | 35098 | 35181 | 12217 | 12300 | 9.83E-24 |
| 1 | 4 | F | 85 | 90.588 | 15407 | 15490 | 24467 | 24551 | 9.83E-24 |
| 1 | 4 | F | 65 | 96.923 | 80553 | 80616 | 10508 | 10571 | 1.27E-22 |
| 1 | 4 | F | 93 | 88.172 | 25280 | 25365 | 12353 | 12443 | 5.91E-21 |
| 1 | 4 | P | 48 | 100 | 24909 | 24956 | 36965 | 37012 | 4.60E-17 |
| 1 | 4 | P | 49 | 97.959 | 41182 | 41230 | 13399 | 13447 | 5.96E-16 |
| 1 | 4 | F | 46 | 95.672 | 100600 | 100645 | 31781 | 31826 | 1.29E-12 |
| 1 | 4 | F | 48 | 93.75 | 49005 | 49052 | 38840 | 38887 | 4.64E-12 |
| 1 | 4 | P | 58 | 87.931 | 34926 | 34983 | 32010 | 32066 | 2.16E-10 |
| 1 | 4 | F | 59 | 86.441 | 25352 | 25410 | 21865 | 21923 | 7.76E-10 |
| 1 | 4 | P | 37 | 97.297 | 85907 | 85943 | 22686 | 22722 | 2.79E-09 |
| 1 | 4 | P | 51 | 88.235 | 35326 | 35376 | 38863 | 38913 | 1.00E-08 |
| 1 | 4 | P | 47 | 89.362 | 22528 | 22574 | 48962 | 49008 | 3.61E-08 |
| 1 | 4 | F | 31 | 100 | 72275 | 72305 | 3410 | 3440 | 1.30E-07 |
| 1 | 4 | P | 37 | 94.595 | 83045 | 83081 | 12357 | 12393 | 1.30E-07 |
| 1 | 4 | F | 33 | 96.97 | 97489 | 97521 | 21711 | 27143 | 4.67E-07 |
| 1 | 4 | P | 30 | 100 | 21913 | 21942 | 50062 | 50091 | 4.67E-07 |
| 1 | 2 | F | 164 | 100 | 98705 | 98868 | 35013 | 35176 | 1.51E-81 |
| 1 | 2 | F | 175 | 92 | 55515 | 55688 | 33826 | 33997 | 3.34E-63 |
| 1 | 2 | F | 116 | 98.276 | 119297 | 119412 | 17397 | 17512 | 1.58E-51 |
| 1 | 2 | P | 126 | 93.651 | 98330 | 98455 | 89599 | 89722 | 1.59E-46 |
| 1 | 2 | F | 89 | 100 | 48964 | 49052 | 57184 | 57272 | 7.44E-40 |
| 1 | 2 | F | 118 | 88.136 | 98174 | 98289 | 34040 | 34150 | 7.54E-30 |
| 1 | 2 | F | 100 | 91 | 20488 | 20581 | 30777 | 30876 | 2.71E-29 |
| 1 | 2 | P | 60 | 100 | 88431 | 88490 | 38586 | 38645 | 9.83E-24 |
| 1 | 2 | P | 69 | 95.652 | 123219 | 123286 | 2655 | 2723 | 3.53E-23 |
| 1 | 2 | P | 65 | 96.923 | 36103 | 36166 | 66765 | 66829 | 1.27E-22 |
| 1 | 2 | F | 60 | 96.667 | 117674 | 117733 | 37692 | 37751 | 2.13E-20 |
| 1 | 2 | F | 66 | 92.424 | 55709 | 55774 | 38722 | 38787 | 9.90E-19 |
| 1 | 2 | P | 46 | 100 | 55572 | 55617 | 24750 | 24795 | 5.96E-16 |
| 1 | 2 | F | 48 | 93.75 | 108154 | 108201 | 57225 | 57272 | 4.64E-12 |
| 1 | 2 | F | 63 | 87.302 | 79913 | 79971 | 85655 | 85717 | 6.00E-11 |
| 1 | 2 | F | 41 | 95.122 | 2776 | 2816 | 47598 | 47638 | 7.76E-10 |
| 1 | 2 | P | 53 | 88.679 | 77767 | 77818 | 62980 | 63032 | 2.79E-09 |
| 1 | 2 | P | 36 | 97.222 | 48051 | 48086 | 78702 | 78737 | 1.00E-08 |
| 1 | 2 | P | 44 | 90.909 | 26385 | 26428 | 22488 | 22531 | 3.61E-08 |
| 1 | 2 | P | 34 | 97.059 | 85907 | 85940 | 37249 | 37282 | 1.30E-07 |
| 1 | 2 | F | 31 | 100 | 57461 | 57491 | 64650 | 64590 | 1.30E-07 |
| 1 | 2 | F | 40 | 92.5 | 85901 | 85940 | 82343 | 82382 | 1.30E-07 |
| 1 | 2 | P | 30 | 100 | 85736 | 85765 | 16702 | 16731 | 4.67E-07 |
| 1 | 2 | P | 30 | 100 | 21913 | 21942 | 17476 | 17505 | 4.67E-07 |
| 1 | 2 | P | 33 | 96.97 | 29246 | 29278 | 33961 | 33993 | 4.67E-07 |
| 1 | 3 | F | 213 | 86.854 | 4628 | 4829 | 28462 | 28671 | 3.36E-58 |
| 1 | 3 | F | 129 | 95.349 | 37401 | 37527 | 20049 | 20177 | 1.58E-51 |
| 1 | 3 | P | 92 | 90.217 | 22292 | 22383 | 28554 | 28645 | 1.63E-26 |
| 1 | 3 | P | 133 | 83.459 | 67315 | 67443 | 28445 | 28576 | 5.87E-26 |
| 1 | 3 | F | 59 | 100 | 17272 | 17330 | 61707 | 61765 | 3.53E-23 |
| 1 | 3 | P | 41 | 100 | 118303 | 118343 | 108 | 148 | 3.58E-13 |
| 1 | 3 | P | 45 | 95.556 | 81391 | 81435 | 32604 | 32648 | 4.64E-12 |
| 1 | 3 | P | 40 | 97.5 | 51393 | 51432 | 40736 | 40775 | 6.00E-11 |
| 1 | 3 | F | 41 | 95.122 | 843 | 883 | 27920 | 27960 | 7.76E-10 |
| 1 | 3 | P | 34 | 100 | 26454 | 26487 | 50660 | 50693 | 2.79E-09 |
| 1 | 3 | F | 65 | 84.615 | 25321 | 25382 | 13311 | 13370 | 1.30E-07 |
| 1 | 3 | P | 39 | 92.308 | 85892 | 85930 | 24557 | 24595 | 4.67E-07 |
| 1 | 5 | F | 97 | 97.938 | 119043 | 119139 | 11637 | 11733 | 5.75E-41 |
| 1 | 5 | F | 88 | 97.727 | 881 | 968 | 9058 | 9144 | 2.08E-35 |
| 1 | 5 | F | 61 | 98.361 | 35234 | 35293 | 17287 | 17347 | 4.57E-22 |
| 1 | 5 | F | 62 | 96.774 | 1890 | 1951 | 9136 | 9197 | 1.64E-21 |
| 1 | 5 | P | 41 | 100 | 118303 | 118343 | 108 | 148 | 3.58E-13 |
| 1 | 5 | P | 41 | 97.561 | 55241 | 55281 | 15960 | 16000 | 1.67E-11 |
| 1 | 5 | F | 67 | 82.09 | 758 | 824 | 9075 | 9141 | 1.30E-07 |
| 1 | 6 | P | 71 | 92.958 | 21769 | 21837 | 3078 | 3148 | 5.91E-21 |
| 1 | 6 | P | 57 | 92.982 | 22439 | 22495 | 301 | 354 | 2.77E-14 |
| 1 | 6 | F | 98 | 80.612 | 264 | 361 | 1899 | 1996 | 3.58E-13 |
| 1 | 6 | P | 49 | 91.837 | 21804 | 21852 | 9622 | 9669 | 2.16E-10 |
| 1 | 6 | F | 35 | 97.143 | 55709 | 55743 | 9374 | 9408 | 3.61E-08 |
| 2 | 2 | P | 114 | 97.368 | 82270 | 82383 | 37248 | 37361 | 7.01E-49 |
| 2 | 2 | F | 88 | 100 | 88556 | 88643 | 51484 | 51571 | 1.98E-39 |
| 2 | 2 | F | 57 | 96.491 | 8586 | 8642 | 6462 | 6517 | 2.63E-18 |
| 2 | 2 | F | 41 | 97.561 | 84691 | 84731 | 10303 | 10343 | 1.23E-11 |
| 2 | 2 | P | 36 | 100 | 83764 | 83799 | 34032 | 34067 | 1.59E-10 |
| 2 | 2 | F | 35 | 97.143 | 37347 | 37381 | 16702 | 16735 | 9.59E-08 |
| 2 | 2 | P | 46 | 89.13 | 33880 | 33925 | 24750 | 24795 | 9.59E-08 |
| 2 | 2 | P | 54 | 85.185 | 3354 | 3407 | 3354 | 3407 | 3.45E-07 |
| 2 | 4 | P | 318 | 100 | 82065 | 82382 | 22689 | 23006 | 2.75E-167 |
| 2 | 4 | F | 116 | 98.276 | 17397 | 17512 | 49983 | 50098 | 1.16E-51 |
| 2 | 4 | F | 113 | 97.345 | 37249 | 37361 | 22689 | 22801 | 2.52E-48 |
| 2 | 4 | F | 78 | 100 | 38660 | 38737 | 12039 | 12116 | 7.16E-34 |
| 2 | 4 | P | 69 | 95.652 | 2655 | 2723 | 53905 | 53972 | 2.61E-23 |
| 2 | 4 | F | 60 | 96.667 | 37692 | 37751 | 48360 | 48419 | 1.57E-20 |
| 2 | 4 | P | 78 | 84.615 | 22978 | 23055 | 27004 | 27076 | 3.43E-12 |
| 2 | 4 | F | 48 | 93.75 | 57225 | 57272 | 38840 | 38887 | 3.43E-12 |
| 2 | 4 | P | 35 | 97.143 | 15499 | 15533 | 21817 | 21851 | 2.67E-08 |
| 2 | 4 | P | 41 | 92.683 | 82104 | 82143 | 5687 | 5726 | 9.59E-08 |
| 2 | 4 | F | 42 | 90.476 | 86145 | 86186 | 9870 | 9911 | 3.45E-07 |
| 2 | 4 | F | 37 | 94.595 | 82354 | 82389 | 21867 | 21902 | 3.45E-07 |
| 2 | 1 | P | 129 | 93.023 | 89599 | 89725 | 98327 | 98455 | 1.17E-46 |
| 2 | 1 | P | 75 | 93.333 | 66765 | 66839 | 36095 | 36166 | 9.39E-23 |
| 2 | 1 | P | 41 | 92.683 | 22491 | 22531 | 26385 | 26425 | 2.67E-08 |
| 2 | 1 | P | 40 | 92.5 | 37249 | 37288 | 85901 | 85940 | 9.59E-08 |
| 2 | 5 | F | 462 | 75.974 | 14817 | 15260 | 21550 | 21996 | 9.00E-53 |
| 2 | 5 | F | 211 | 74.882 | 16089 | 16298 | 22445 | 22651 | 9.46E-18 |
| 2 | 5 | F | 97 | 81.443 | 16776 | 16872 | 23112 | 23208 | 2.05E-14 |
| 2 | 5 | P | 39 | 97.436 | 15733 | 15770 | 14962 | 15000 | 5.73E-10 |
| 2 | 5 | F | 69 | 82.609 | 17185 | 17253 | 23390 | 23458 | 7.42E-09 |
| 2 | 3 | F | 109 | 99.083 | 18368 | 18476 | 27124 | 27232 | 1.95E-49 |
| 2 | 3 | F | 81 | 97.531 | 72105 | 72185 | 60383 | 60463 | 3.33E-32 |
| 2 | 3 | F | 77 | 87.013 | 35995 | 36062 | 55328 | 55404 | 7.36E-14 |
| 2 | 3 | F | 38 | 97.368 | 37302 | 37339 | 13296 | 13333 | 5.73E-10 |
| 2 | 3 | F | 44 | 93.182 | 38928 | 38971 | 63583 | 63626 | 5.73E-10 |
| 2 | 3 | P | 40 | 95 | 82290 | 82329 | 13296 | 13335 | 2.06E-09 |
| 2 | 3 | P | 33 | 100 | 85070 | 85102 | 13236 | 13268 | 7.42E-09 |
| 2 | 3 | F | 35 | 97.143 | 37204 | 37238 | 13150 | 13184 | 2.67E-08 |
| 2 | 6 | P | 47 | 95.745 | 32113 | 32159 | 9343 | 9389 | 2.65E-13 |
| 2 | 6 | F | 42 | 95.238 | 17671 | 17711 | 3072 | 3113 | 5.73E-10 |
| 2 | 6 | F | 38 | 97.368 | 38719 | 38756 | 9371 | 9408 | 5.73E-10 |
| 3 | 3 | F | 68 | 97.059 | 55774 | 55841 | 26328 | 26394 | 1.47E-24 |
| 3 | 3 | P | 61 | 90.164 | 14442 | 14500 | 6836 | 6896 | 5.38E-14 |
| 3 | 3 | P | 57 | 91.228 | 6843 | 6899 | 14439 | 14495 | 5.38E-14 |
| 3 | 3 | P | 44 | 97.727 | 66756 | 66799 | 19475 | 19517 | 6.96E-13 |
| 3 | 3 | P | 44 | 95.455 | 39434 | 39477 | 10431 | 10474 | 9.00E-12 |
| 3 | 3 | F | 54 | 90.741 | 42933 | 42985 | 25803 | 25856 | 9.00E-12 |
| 3 | 3 | F | 43 | 95.349 | 53209 | 53251 | 16413 | 16454 | 1.16E-10 |
| 3 | 3 | F | 47 | 91.489 | 31951 | 31997 | 31822 | 31868 | 4.19E-10 |
| 3 | 3 | F | 35 | 94.286 | 31908 | 31942 | 31851 | 31885 | 9.06E-07 |
| 3 | 3 | F | 32 | 96.875 | 54441 | 54472 | 45004 | 45035 | 9.06E-07 |
| 3 | 5 | F | 2073 | 100 | 1 | 2073 | 1 | 2073 | 0 |
| 3 | 5 | P | 84 | 89.286 | 305 | 387 | 9725 | 9806 | 3.19E-21 |
| 3 | 4 | P | 137 | 97.08 | 25764 | 25900 | 7071 | 7207 | 3.90E-60 |
| 3 | 4 | F | 62 | 96.774 | 149 | 210 | 15374 | 15435 | 8.87E-22 |
| 3 | 4 | P | 41 | 100 | 108 | 148 | 48989 | 49029 | 1.93E-13 |
| 3 | 4 | P | 44 | 95.455 | 55785 | 55827 | 13278 | 13321 | 3.24E-11 |
| 3 | 4 | P | 44 | 95.455 | 26339 | 26381 | 13278 | 13321 | 3.24E-11 |
| 3 | 4 | P | 39 | 97.436 | 42933 | 42971 | 7130 | 7168 | 1.16E-10 |
| 3 | 4 | F | 51 | 90.196 | 55730 | 55780 | 13223 | 13273 | 1.16E-10 |
| 3 | 4 | F | 40 | 95 | 13296 | 13335 | 22742 | 22781 | 1.51E-09 |
| 3 | 4 | P | 45 | 91.111 | 55785 | 55828 | 23004 | 23046 | 7.00E-08 |
| 3 | 4 | P | 45 | 91.111 | 26339 | 26382 | 23004 | 23046 | 7.00E-08 |
| 3 | 4 | P | 35 | 94.286 | 55792 | 55826 | 6886 | 6920 | 9.06E-07 |
| 3 | 4 | P | 35 | 94.286 | 26346 | 26380 | 6886 | 6920 | 9.06E-07 |
| 3 | 1 | P | 89 | 91.011 | 28557 | 28645 | 22292 | 22380 | 8.81E-27 |
| 3 | 1 | P | 32 | 96.875 | 32604 | 32635 | 48077 | 48108 | 9.06E-07 |
| 3 | 2 | F | 56 | 85.714 | 6854 | 6909 | 37351 | 37401 | 9.06E-07 |
| 3 | 2 | P | 70 | 81.429 | 52519 | 52588 | 37686 | 37752 | 9.06E-07 |
| 3 | 2 | P | 74 | 81.081 | 24542 | 24614 | 82315 | 82383 | 9.06E-07 |
| 3 | 6 | F | 44 | 95.455 | 26339 | 26381 | 921 | 964 | 3.24E-11 |
| 3 | 6 | F | 44 | 95.455 | 55785 | 55827 | 921 | 964 | 3.24E-11 |
| 3 | 6 | P | 30 | 100 | 57247 | 57276 | 374 | 403 | 2.52E-07 |
| 4 | 4 | F | 40 | 100 | 13315 | 13354 | 12449 | 12488 | 5.81E-13 |
| 4 | 4 | F | 38 | 94.737 | 23009 | 23046 | 13284 | 13321 | 1.63E-08 |
| 4 | 4 | F | 41 | 92.683 | 22928 | 22967 | 5687 | 5726 | 5.85E-08 |
| 4 | 4 | P | 30 | 100 | 22688 | 22717 | 21867 | 21896 | 2.10E-07 |
| 4 | 4 | F | 36 | 94.444 | 13279 | 13314 | 6886 | 6920 | 7.56E-07 |
| 4 | 1 | P | 87 | 89.655 | 12217 | 12303 | 35095 | 35181 | 4.42E-24 |
| 4 | 1 | P | 43 | 93.023 | 22686 | 22728 | 85901 | 85943 | 1.26E-09 |
| 4 | 2 | P | 75 | 85.333 | 27007 | 27076 | 22978 | 23052 | 2.09E-12 |
| 4 | 2 | P | 29 | 100 | 21867 | 21895 | 37249 | 37277 | 7.56E-07 |
| 4 | 7 | F | 889 | 74.128 | 20835 | 21693 | 518 | 1381 | 8.74E-86 |
| 4 | 7 | F | 265 | 78.868 | 22074 | 22335 | 1408 | 1671 | 1.55E-43 |
| 4 | 7 | F | 61 | 86.885 | 20589 | 20649 | 313 | 373 | 2.70E-11 |
| 4 | 7 | F | 51 | 88.235 | 22446 | 22496 | 1735 | 1785 | 4.52E-09 |
| 4 | 3 | P | 38 | 92.105 | 6886 | 6923 | 26343 | 26380 | 7.56E-07 |
| 4 | 3 | P | 38 | 92.105 | 6886 | 6923 | 55789 | 55826 | 7.56E-07 |
| 4 | 5 | F | 97 | 97.938 | 49729 | 49825 | 11637 | 11733 | 2.59E-41 |
| 4 | 5 | F | 62 | 96.774 | 15374 | 15435 | 149 | 210 | 7.40E-22 |
| 4 | 5 | P | 41 | 100 | 48989 | 49029 | 108 | 148 | 1.61E-13 |
| 4 | 6 | P | 57 | 100 | 13265 | 13321 | 921 | 977 | 2.06E-22 |
| 4 | 6 | P | 44 | 95.455 | 23009 | 23052 | 915 | 958 | 7.51E-12 |
| 4 | 6 | P | 39 | 92.308 | 6886 | 6923 | 925 | 963 | 7.56E-07 |
| 5 | 5 | P | 84 | 89.286 | 9725 | 9806 | 305 | 387 | 1.13E-21 |
| 6 | 6 | F | 105 | 86.667 | 9516 | 9620 | 2967 | 3071 | 1.80E-26 |
| 6 | 4 | P | 36 | 94.444 | 928 | 963 | 6886 | 6920 | 1.43E-07 |
| 6 | 4 | P | 37 | 91.892 | 2955 | 2991 | 20621 | 20657 | 5.14E-07 |
| 6 | 1 | P | 58 | 98.276 | 3094 | 3151 | 21766 | 21823 | 5.03E-22 |
| 6 | 3 | F | 38 | 92.105 | 6429 | 6464 | 66740 | 66777 | 5.14E-07 |

Note: Chr: chromosome number; type: type of repeat sequence; P is reverse complementary repeat, F is forward repeat; Aignment length: length after alignment; Similarity: the similarity of repeated sequences; start and end are the starting point and end point of different repeat sequences respectively.

**Table S4.** **The RNA editing events prediction in *P. granatum* mitogenome**

| ID | Gene | Nt Pos | AA Pos | Align Col | Effect | Score |
| --- | --- | --- | --- | --- | --- | --- |
| Punica_granatum_1 | atp1 | 1490 | 497 | 499 | CCA (P) => CTA (L) | 0.9 |
| Punica_granatum_1 | atp1 | 1523 | 508 | 510 | TCC (S) => TTC (F) | 0.3 |
| Punica_granatum_1 | atp1 | 1528 | 510 | 512 | CAA (Q) => TAA (X) | 0.56 |
| Punica_granatum_1 | atp4 | 56 | 19 | 23 | CCA (P) => CTA (L) | 0.86 |
| Punica_granatum_1 | atp4 | 59 | 20 | 24 | TCT (S) => TTT (F) | 0.57 |
| Punica_granatum_1 | atp4 | 89 | 30 | 34 | TCA (S) => TTA (L) | 1 |
| Punica_granatum_1 | atp4 | 118 | 40 | 44 | CGT (R) => TGT (C) | 0.71 |
| Punica_granatum_1 | atp4 | 215 | 72 | 76 | TCG (S) => TTG (L) | 1 |
| Punica_granatum_1 | atp4 | 226 | 76 | 80 | CCC (P) => TTC (F) | 0.57 |
| Punica_granatum_1 | atp4 | 227 | 76 | 80 | CCC (P) => TTC (F) | 0.57 |
| Punica_granatum_1 | atp4 | 248 | 83 | 87 | CCT (P) => CTT (L) | 1 |
| Punica_granatum_1 | atp4 | 251 | 84 | 88 | CCG (P) => CTG (L) | 0.43 |
| Punica_granatum_1 | atp4 | 395 | 132 | 140 | TCA (S) => TTA (L) | 1 |
| Punica_granatum_1 | atp4 | 407 | 136 | 144 | CCA (P) => CTA (L) | 0.71 |
| Punica_granatum_1 | atp4 | 416 | 139 | 147 | ACT (T) => ATT (I) | 0.86 |
| Punica_granatum_1 | atp4 | 490 | 164 | 172 | CCG (P) => TCG (S) | 0.57 |
| Punica_granatum_1 | atp8 | 47 | 16 | 16 | TCA (S) => TTA (L) | 1 |
| Punica_granatum_1 | atp8 | 58 | 20 | 20 | CTC (L) => TTC (F) | 1 |
| Punica_granatum_1 | atp8 | 77 | 26 | 26 | CCC (P) => CTC (L) | 0.38 |
| Punica_granatum_1 | atp8 | 452 | 151 | 155 | CCA (P) => CTA (L) | 0.75 |
| Punica_granatum_1 | atp9 | 20 | 7 | 7 | TCA (S) => TTA (L) | 1 |
| Punica_granatum_1 | atp9 | 50 | 17 | 17 | TCA (S) => TTA (L) | 1 |
| Punica_granatum_1 | atp9 | 134 | 45 | 45 | TCA (S) => TTA (L) | 1 |
| Punica_granatum_1 | atp9 | 191 | 64 | 64 | CCA (P) => CTA (L) | 1 |
| Punica_granatum_1 | atp9 | 223 | 75 | 75 | CGA (R) => TGA (X) | 1 |
| Punica_granatum_1 | ccmC | 76 | 26 | 32 | CGG (R) => TGG (W) | 0.78 |
| Punica_granatum_1 | ccmC | 103 | 35 | 41 | CAT (H) => TAT (Y) | 1 |
| Punica_granatum_1 | ccmC | 115 | 39 | 45 | CGG (R) => TGG (W) | 0.78 |
| Punica_granatum_1 | ccmC | 161 | 54 | 60 | CCG (P) => CTG (L) | 0.78 |
| Punica_granatum_1 | ccmC | 179 | 60 | 66 | GCG (A) => GTG (V) | 0.78 |
| Punica_granatum_1 | ccmC | 184 | 62 | 68 | CGG (R) => TGG (W) | 1 |
| Punica_granatum_1 | ccmC | 331 | 111 | 117 | CGG (R) => TGG (W) | 1 |
| Punica_granatum_1 | ccmC | 395 | 132 | 138 | TCG (S) => TTG (L) | 1 |
| Punica_granatum_1 | ccmC | 400 | 134 | 140 | CTT (L) => TTT (F) | 0.89 |
| Punica_granatum_1 | ccmC | 436 | 146 | 152 | CCT (P) => TCT (S) | 0.89 |
| Punica_granatum_1 | ccmC | 446 | 149 | 155 | CCG (P) => CTG (L) | 0.78 |
| Punica_granatum_1 | ccmC | 458 | 153 | 159 | TCA (S) => TTA (L) | 0.78 |
| Punica_granatum_1 | ccmC | 463 | 155 | 161 | CGT (R) => TGT (C) | 1 |
| Punica_granatum_1 | ccmC | 467 | 156 | 162 | GCT (A) => GTT (V) | 0.78 |
| Punica_granatum_1 | ccmC | 473 | 158 | 164 | CCG (P) => CTG (L) | 1 |
| Punica_granatum_1 | ccmC | 497 | 166 | 172 | TCT (S) => TTT (F) | 1 |
| Punica_granatum_1 | ccmC | 521 | 174 | 180 | TCG (S) => TTG (L) | 1 |
| Punica_granatum_1 | ccmC | 548 | 183 | 189 | TCT (S) => TTT (F) | 1 |
| Punica_granatum_1 | ccmC | 568 | 190 | 196 | CCT (P) => TCT (S) | 1 |
| Punica_granatum_1 | ccmC | 575 | 192 | 198 | CCC (P) => CTC (L) | 1 |
| Punica_granatum_1 | ccmC | 605 | 202 | 208 | TCC (S) => TTC (F) | 1 |
| Punica_granatum_1 | ccmC | 608 | 203 | 209 | CCC (P) => CTC (L) | 0.89 |
| Punica_granatum_1 | ccmC | 614 | 205 | 211 | TCA (S) => TTA (L) | 0.78 |
| Punica_granatum_1 | ccmC | 619 | 207 | 213 | CGT (R) => TGT (C) | 0.78 |
| Punica_granatum_1 | ccmC | 650 | 217 | 223 | CCT (P) => CTT (L) | 0.78 |
| Punica_granatum_1 | ccmC | 665 | 222 | 228 | CCC (P) => CTC (L) | 0.78 |
| Punica_granatum_1 | ccmFn | 38 | 13 | 15 | CCG (P) => CTG (L) | 1 |
| Punica_granatum_1 | ccmFn | 98 | 33 | 35 | CCC (P) => CTC (L) | 1 |
| Punica_granatum_1 | ccmFn | 137 | 46 | 48 | TCG (S) => TTG (L) | 1 |
| Punica_granatum_1 | ccmFn | 142 | 48 | 50 | CGT (R) => TGT (C) | 1 |
| Punica_granatum_1 | ccmFn | 151 | 51 | 53 | CCG (P) => TCG (S) | 0.83 |
| Punica_granatum_1 | ccmFn | 248 | 83 | 85 | TCA (S) => TTA (L) | 1 |
| Punica_granatum_1 | ccmFn | 256 | 86 | 88 | CGG (R) => TGG (W) | 1 |
| Punica_granatum_1 | ccmFn | 263 | 88 | 90 | CCA (P) => CTA (L) | 0.83 |
| Punica_granatum_1 | ccmFn | 283 | 95 | 97 | CTT (L) => TTT (F) | 0.83 |
| Punica_granatum_1 | ccmFn | 334 | 112 | 114 | CAT (H) => TAT (Y) | 0.67 |
| Punica_granatum_1 | ccmFn | 356 | 119 | 121 | TCC (S) => TTC (F) | 0.67 |
| Punica_granatum_1 | ccmFn | 478 | 160 | 164 | CGT (R) => TGT (C) | 0.83 |
| Punica_granatum_1 | ccmFn | 559 | 187 | 191 | CCT (P) => TTT (F) | 0.5 |
| Punica_granatum_1 | ccmFn | 560 | 187 | 191 | CCT (P) => TTT (F) | 0.5 |
| Punica_granatum_1 | ccmFn | 706 | 236 | 240 | CCT (P) => TTT (F) | 0.67 |
| Punica_granatum_1 | ccmFn | 707 | 236 | 240 | CCT (P) => TTT (F) | 0.67 |
| Punica_granatum_1 | ccmFn | 716 | 239 | 243 | TCA (S) => TTA (L) | 0.83 |
| Punica_granatum_1 | ccmFn | 754 | 252 | 256 | CGT (R) => TGT (C) | 1 |
| Punica_granatum_1 | ccmFn | 776 | 259 | 263 | TCA (S) => TTA (L) | 1 |
| Punica_granatum_1 | ccmFn | 788 | 263 | 267 | CCA (P) => CTA (L) | 1 |
| Punica_granatum_1 | ccmFn | 803 | 268 | 272 | TCA (S) => TTA (L) | 1 |
| Punica_granatum_1 | ccmFn | 952 | 318 | 322 | CGT (R) => TGT (C) | 1 |
| Punica_granatum_1 | ccmFn | 1270 | 424 | 446 | CGG (R) => TGG (W) | 1 |
| Punica_granatum_1 | ccmFn | 1298 | 433 | 455 | CCA (P) => CTA (L) | 1 |
| Punica_granatum_1 | ccmFn | 1315 | 439 | 461 | CAT (H) => TAT (Y) | 1 |
| Punica_granatum_1 | ccmFn | 1330 | 444 | 466 | CGG (R) => TGG (W) | 1 |
| Punica_granatum_1 | ccmFn | 1348 | 450 | 472 | CGG (R) => TGG (W) | 1 |
| Punica_granatum_1 | ccmFn | 1381 | 461 | 483 | CGG (R) => TGG (W) | 1 |
| Punica_granatum_1 | ccmFn | 1399 | 467 | 489 | CGT (R) => TGT (C) | 1 |
| Punica_granatum_1 | ccmFn | 1442 | 481 | 503 | TCG (S) => TTG (L) | 1 |
| Punica_granatum_1 | ccmFn | 1462 | 488 | 510 | CTT (L) => TTT (F) | 1 |
| Punica_granatum_1 | ccmFn | 1561 | 521 | 543 | CGG (R) => TGG (W) | 0.67 |
| Punica_granatum_1 | cob | 286 | 96 | 97 | CTC (L) => TTC (F) | 1 |
| Punica_granatum_1 | cob | 298 | 100 | 101 | CAC (H) => TAC (Y) | 1 |
| Punica_granatum_1 | cob | 325 | 109 | 110 | CAT (H) => TAT (Y) | 1 |
| Punica_granatum_1 | cob | 358 | 120 | 121 | CGG (R) => TGG (W) | 1 |
| Punica_granatum_1 | cob | 407 | 136 | 137 | ACA (T) => ATA (I) | 1 |
| Punica_granatum_1 | cob | 568 | 190 | 191 | CAT (H) => TAT (Y) | 0.92 |
| Punica_granatum_1 | cob | 853 | 285 | 286 | CAT (H) => TAT (Y) | 1 |
| Punica_granatum_1 | cob | 908 | 303 | 304 | CCA (P) => CTA (L) | 1 |
| Punica_granatum_1 | cob | 982 | 328 | 329 | CAC (H) => TAC (Y) | 0.85 |
| Punica_granatum_1 | cob | 1084 | 362 | 363 | CCT (P) => TCT (S) | 1 |
| Punica_granatum_1 | cox3 | 112 | 38 | 39 | CCA (P) => TCA (S) | 1 |
| Punica_granatum_1 | cox3 | 304 | 102 | 103 | CGG (R) => TGG (W) | 1 |
| Punica_granatum_1 | cox3 | 311 | 104 | 105 | TCT (S) => TTT (F) | 0.92 |
| Punica_granatum_1 | cox3 | 413 | 138 | 139 | CCT (P) => CTT (L) | 0.83 |
| Punica_granatum_1 | cox3 | 512 | 171 | 172 | TCA (S) => TTA (L) | 0.75 |
| Punica_granatum_1 | cox3 | 565 | 189 | 190 | CCC (P) => TTC (F) | 0.92 |
| Punica_granatum_1 | cox3 | 566 | 189 | 190 | CCC (P) => TTC (F) | 0.92 |
| Punica_granatum_1 | cox3 | 653 | 218 | 219 | TCG (S) => TTG (L) | 1 |
| Punica_granatum_1 | cox3 | 754 | 252 | 253 | CGG (R) => TGG (W) | 0.92 |
| Punica_granatum_1 | nad1 | 2 | 1 | 4 | ACG (T) => ATG (M) | 0.8 |
| Punica_granatum_1 | nad1 | 167 | 56 | 59 | TCG (S) => TTG (L) | 1 |
| Punica_granatum_1 | nad1 | 215 | 72 | 75 | TCC (S) => TTC (F) | 0.9 |
| Punica_granatum_1 | nad1 | 265 | 89 | 92 | CGG (R) => TGG (W) | 1 |
| Punica_granatum_1 | nad1 | 308 | 103 | 106 | CCG (P) => CTG (L) | 1 |
| Punica_granatum_1 | nad1 | 368 | 123 | 126 | ACA (T) => ATA (I) | 0.7 |
| Punica_granatum_1 | nad1 | 376 | 126 | 129 | CGG (R) => TGG (W) | 1 |
| Punica_granatum_1 | nad1 | 490 | 164 | 167 | CCC (P) => TCC (S) | 1 |
| Punica_granatum_1 | nad1 | 493 | 165 | 168 | CGT (R) => TGT (C) | 1 |
| Punica_granatum_1 | nad1 | 500 | 167 | 170 | TCG (S) => TTG (L) | 0.7 |
| Punica_granatum_1 | nad1 | 536 | 179 | 182 | TCC (S) => TTC (F) | 1 |
| Punica_granatum_1 | nad1 | 580 | 194 | 197 | CGT (R) => TGT (C) | 1 |
| Punica_granatum_1 | nad1 | 635 | 212 | 215 | TCA (S) => TTA (L) | 1 |
| Punica_granatum_1 | nad1 | 674 | 225 | 228 | TCT (S) => TTT (F) | 0.9 |
| Punica_granatum_1 | nad1 | 734 | 245 | 248 | TCG (S) => TTG (L) | 0.9 |
| Punica_granatum_1 | nad1 | 740 | 247 | 250 | TCT (S) => TTT (F) | 1 |
| Punica_granatum_1 | nad1 | 743 | 248 | 251 | CCA (P) => CTA (L) | 1 |
| Punica_granatum_1 | nad1 | 755 | 252 | 255 | CCG (P) => CTG (L) | 1 |
| Punica_granatum_1 | nad1 | 823 | 275 | 278 | CTC (L) => TTC (F) | 0.7 |
| Punica_granatum_1 | nad1 | 898 | 300 | 303 | CGG (R) => TGG (W) | 1 |
| Punica_granatum_1 | nad1 | 928 | 310 | 313 | CGG (R) => TGG (W) | 1 |
| Punica_granatum_1 | nad1 | 937 | 313 | 316 | CCT (P) => TCT (S) | 0.6 |
| Punica_granatum_1 | nad2 | 26 | 9 | 10 | TCC (S) => TTC (F) | 0.89 |
| Punica_granatum_1 | nad2 | 262 | 88 | 89 | CTT (L) => TTT (F) | 0.56 |
| Punica_granatum_1 | nad2 | 356 | 119 | 120 | CCA (P) => CTA (L) | 1 |
| Punica_granatum_1 | nad2 | 361 | 121 | 122 | CCT (P) => TCT (S) | 1 |
| Punica_granatum_1 | nad2 | 367 | 123 | 124 | CGC (R) => TGC (C) | 1 |
| Punica_granatum_1 | nad2 | 394 | 132 | 133 | CAT (H) => TAT (Y) | 1 |
| Punica_granatum_1 | nad2 | 401 | 134 | 135 | TCA (S) => TTA (L) | 1 |
| Punica_granatum_1 | nad2 | 428 | 143 | 144 | CCT (P) => CTT (L) | 1 |
| Punica_granatum_1 | nad2 | 497 | 166 | 167 | TCG (S) => TTG (L) | 1 |
| Punica_granatum_1 | nad2 | 523 | 175 | 176 | CCC (P) => TCC (S) | 1 |
| Punica_granatum_1 | nad2 | 788 | 263 | 267 | TCT (S) => TTT (F) | 0.67 |
| Punica_granatum_1 | nad2 | 800 | 267 | 271 | TCA (S) => TTA (L) | 1 |
| Punica_granatum_1 | nad2 | 809 | 270 | 274 | TCT (S) => TTT (F) | 1 |
| Punica_granatum_1 | nad2 | 920 | 307 | 311 | CCT (P) => CTT (L) | 1 |
| Punica_granatum_1 | nad2 | 928 | 310 | 314 | CAT (H) => TAT (Y) | 1 |
| Punica_granatum_1 | nad2 | 958 | 320 | 324 | CGT (R) => TGT (C) | 0.78 |
| Punica_granatum_1 | nad2 | 962 | 321 | 325 | ACT (T) => ATT (I) | 1 |
| Punica_granatum_1 | nad2 | 1058 | 353 | 357 | TCA (S) => TTA (L) | 1 |
| Punica_granatum_1 | nad2 | 1127 | 376 | 380 | TCG (S) => TTG (L) | 1 |
| Punica_granatum_1 | nad2 | 1247 | 416 | 420 | CCA (P) => CTA (L) | 0.67 |
| Punica_granatum_1 | nad2 | 1298 | 433 | 437 | GCG (A) => GTG (V) | 1 |
| Punica_granatum_1 | nad2 | 1400 | 467 | 471 | TCA (S) => TTA (L) | 0.67 |
| Punica_granatum_1 | nad2 | 1403 | 468 | 472 | TCC (S) => TTC (F) | 1 |
| Punica_granatum_1 | nad2 | 1457 | 486 | 490 | TCA (S) => TTA (L) | 1 |
| Punica_granatum_1 | nad3 | 5 | 2 | 2 | TCA (S) => TTA (L) | 0.79 |
| Punica_granatum_1 | nad3 | 23 | 8 | 8 | TCT (S) => TTT (F) | 0.23 |
| Punica_granatum_1 | nad3 | 44 | 15 | 15 | CCG (P) => CTG (L) | 1 |
| Punica_granatum_1 | nad3 | 80 | 27 | 27 | CCA (P) => CTA (L) | 1 |
| Punica_granatum_1 | nad3 | 146 | 49 | 50 | TCC (S) => TTC (F) | 1 |
| Punica_granatum_1 | nad3 | 208 | 70 | 71 | CCT (P) => TTT (F) | 0.95 |
| Punica_granatum_1 | nad3 | 209 | 70 | 71 | CCT (P) => TTT (F) | 0.95 |
| Punica_granatum_1 | nad3 | 215 | 72 | 73 | CCG (P) => CTG (L) | 1 |
| Punica_granatum_1 | nad3 | 247 | 83 | 84 | CCT (P) => TCT (S) | 1 |
| Punica_granatum_1 | nad3 | 251 | 84 | 85 | CCC (P) => CTC (L) | 0.91 |
| Punica_granatum_1 | nad3 | 266 | 89 | 90 | CCG (P) => CTG (L) | 1 |
| Punica_granatum_1 | nad3 | 275 | 92 | 93 | TCT (S) => TTT (F) | 1 |
| Punica_granatum_1 | nad3 | 317 | 106 | 107 | TCT (S) => TTT (F) | 0.95 |
| Punica_granatum_1 | nad3 | 344 | 115 | 116 | TCG (S) => TTG (L) | 1 |
| Punica_granatum_1 | nad3 | 349 | 117 | 118 | CGG (R) => TGG (W) | 1 |
| Punica_granatum_1 | nad4 | 29 | 10 | 10 | TCT (S) => TTT (F) | 0.67 |
| Punica_granatum_1 | nad4 | 74 | 25 | 25 | ACT (T) => ATT (I) | 0.89 |
| Punica_granatum_1 | nad4 | 77 | 26 | 26 | CCT (P) => CTT (L) | 0.78 |
| Punica_granatum_1 | nad4 | 107 | 36 | 36 | CCG (P) => CTG (L) | 1 |
| Punica_granatum_1 | nad4 | 154 | 52 | 52 | CCC (P) => TCC (S) | 1 |
| Punica_granatum_1 | nad4 | 164 | 55 | 55 | CCT (P) => CTT (L) | 0.67 |
| Punica_granatum_1 | nad4 | 166 | 56 | 56 | CGG (R) => TGG (W) | 1 |
| Punica_granatum_1 | nad4 | 197 | 66 | 66 | TCT (S) => TTT (F) | 1 |
| Punica_granatum_1 | nad4 | 362 | 121 | 121 | ACA (T) => ATA (I) | 0.89 |
| Punica_granatum_1 | nad4 | 368 | 123 | 123 | TCT (S) => TTT (F) | 1 |
| Punica_granatum_1 | nad4 | 376 | 126 | 126 | CGT (R) => TGT (C) | 0.78 |
| Punica_granatum_1 | nad4 | 403 | 135 | 135 | CGC (R) => TGC (C) | 1 |
| Punica_granatum_1 | nad4 | 416 | 139 | 139 | CCT (P) => CTT (L) | 0.89 |
| Punica_granatum_1 | nad4 | 433 | 145 | 145 | CTT (L) => TTT (F) | 1 |
| Punica_granatum_1 | nad4 | 436 | 146 | 146 | CCC (P) => TTC (F) | 0.89 |
| Punica_granatum_1 | nad4 | 437 | 146 | 146 | CCC (P) => TTC (F) | 0.89 |
| Punica_granatum_1 | nad4 | 449 | 150 | 150 | CCA (P) => CTA (L) | 1 |
| Punica_granatum_1 | nad4 | 577 | 193 | 193 | CTC (L) => TTC (F) | 0.67 |
| Punica_granatum_1 | nad4 | 608 | 203 | 203 | TCA (S) => TTA (L) | 1 |
| Punica_granatum_1 | nad4 | 659 | 220 | 220 | TCT (S) => TTT (F) | 1 |
| Punica_granatum_1 | nad4 | 767 | 256 | 256 | CCT (P) => CTT (L) | 1 |
| Punica_granatum_1 | nad4 | 836 | 279 | 279 | TCC (S) => TTC (F) | 1 |
| Punica_granatum_1 | nad4 | 857 | 286 | 286 | CCA (P) => CTA (L) | 1 |
| Punica_granatum_1 | nad4 | 896 | 299 | 299 | TCA (S) => TTA (L) | 0.89 |
| Punica_granatum_1 | nad4 | 977 | 326 | 326 | CCG (P) => CTG (L) | 0.78 |
| Punica_granatum_1 | nad4 | 1007 | 336 | 336 | CCA (P) => CTA (L) | 1 |
| Punica_granatum_1 | nad4 | 1010 | 337 | 337 | CCG (P) => CTG (L) | 1 |
| Punica_granatum_1 | nad4 | 1109 | 370 | 370 | TCA (S) => TTA (L) | 1 |
| Punica_granatum_1 | nad4 | 1129 | 377 | 377 | CTC (L) => TTC (F) | 1 |
| Punica_granatum_1 | nad4 | 1132 | 378 | 378 | CCT (P) => TCT (S) | 1 |
| Punica_granatum_1 | nad4 | 1142 | 381 | 381 | TCC (S) => TTC (F) | 1 |
| Punica_granatum_1 | nad4 | 1148 | 383 | 383 | TCT (S) => TTT (F) | 0.89 |
| Punica_granatum_1 | nad4 | 1151 | 384 | 384 | TCC (S) => TTC (F) | 1 |
| Punica_granatum_1 | nad4 | 1172 | 391 | 391 | TCA (S) => TTA (L) | 1 |
| Punica_granatum_1 | nad4 | 1205 | 402 | 402 | CCC (P) => CTC (L) | 1 |
| Punica_granatum_1 | nad4 | 1355 | 452 | 452 | CCA (P) => CTA (L) | 1 |
| Punica_granatum_1 | nad4 | 1373 | 458 | 458 | TCC (S) => TTC (F) | 0.78 |
| Punica_granatum_1 | nad4 | 1417 | 473 | 473 | CAC (H) => TAC (Y) | 1 |
| Punica_granatum_1 | nad4 | 1433 | 478 | 478 | CCG (P) => CTG (L) | 1 |
| Punica_granatum_1 | nad4L | 8 | 3 | 3 | CCT (P) => CTT (L) | 0.88 |
| Punica_granatum_1 | nad4L | 41 | 14 | 14 | TCT (S) => TTT (F) | 1 |
| Punica_granatum_1 | nad4L | 55 | 19 | 19 | CGG (R) => TGG (W) | 1 |
| Punica_granatum_1 | nad4L | 86 | 29 | 29 | CCT (P) => CTT (L) | 1 |
| Punica_granatum_1 | nad4L | 95 | 32 | 32 | TCA (S) => TTA (L) | 1 |
| Punica_granatum_1 | nad4L | 100 | 34 | 34 | CCA (P) => TCA (S) | 1 |
| Punica_granatum_1 | nad4L | 110 | 37 | 37 | TCA (S) => TTA (L) | 1 |
| Punica_granatum_1 | nad4L | 158 | 53 | 53 | TCG (S) => TTG (L) | 1 |
| Punica_granatum_1 | nad4L | 179 | 60 | 60 | TCA (S) => TTA (L) | 0.75 |
| Punica_granatum_1 | nad4L | 188 | 63 | 63 | TCA (S) => TTA (L) | 0.88 |
| Punica_granatum_1 | nad4L | 197 | 66 | 66 | CCA (P) => CTA (L) | 0.88 |
| Punica_granatum_1 | nad4L | 281 | 94 | 94 | TCT (S) => TTT (F) | 0.88 |
| Punica_granatum_1 | nad5 | 155 | 52 | 52 | CCG (P) => CTG (L) | 1 |
| Punica_granatum_1 | nad5 | 242 | 81 | 81 | CCG (P) => CTG (L) | 1 |
| Punica_granatum_1 | nad5 | 358 | 120 | 120 | CTT (L) => TTT (F) | 1 |
| Punica_granatum_1 | nad5 | 374 | 125 | 125 | CCA (P) => CTA (L) | 0.9 |
| Punica_granatum_1 | nad5 | 398 | 133 | 133 | TCT (S) => TTT (F) | 0.9 |
| Punica_granatum_1 | nad5 | 494 | 165 | 165 | ACA (T) => ATA (I) | 1 |
| Punica_granatum_1 | nad5 | 506 | 169 | 169 | CCT (P) => CTT (L) | 1 |
| Punica_granatum_1 | nad5 | 539 | 180 | 180 | CCT (P) => CTT (L) | 1 |
| Punica_granatum_1 | nad5 | 548 | 183 | 183 | TCG (S) => TTG (L) | 0.4 |
| Punica_granatum_1 | nad5 | 553 | 185 | 185 | CGT (R) => TGT (C) | 1 |
| Punica_granatum_1 | nad5 | 598 | 200 | 200 | CGT (R) => TGT (C) | 1 |
| Punica_granatum_1 | nad5 | 676 | 226 | 230 | CTT (L) => TTT (F) | 0.9 |
| Punica_granatum_1 | nad5 | 713 | 238 | 242 | TCG (S) => TTG (L) | 1 |
| Punica_granatum_1 | nad5 | 725 | 242 | 246 | TCA (S) => TTA (L) | 1 |
| Punica_granatum_1 | nad5 | 835 | 279 | 283 | CCA (P) => TCA (S) | 0.9 |
| Punica_granatum_1 | nad5 | 863 | 288 | 292 | TCT (S) => TTT (F) | 0.8 |
| Punica_granatum_1 | nad5 | 875 | 292 | 296 | ACG (T) => ATG (M) | 1 |
| Punica_granatum_1 | nad5 | 1400 | 467 | 471 | TCA (S) => TTA (L) | 1 |
| Punica_granatum_1 | nad5 | 1490 | 497 | 501 | CCC (P) => CTC (L) | 0.8 |
| Punica_granatum_1 | nad5 | 1550 | 517 | 521 | ACC (T) => ATC (I) | 0.9 |
| Punica_granatum_1 | nad5 | 1568 | 523 | 527 | CCG (P) => CTG (L) | 0.9 |
| Punica_granatum_1 | nad5 | 1580 | 527 | 531 | TCA (S) => TTA (L) | 0.8 |
| Punica_granatum_1 | nad5 | 1589 | 530 | 534 | TCT (S) => TTT (F) | 0.9 |
| Punica_granatum_1 | nad5 | 1610 | 537 | 541 | CCC (P) => CTC (L) | 0.7 |
| Punica_granatum_1 | nad5 | 1916 | 639 | 643 | TCT (S) => TTT (F) | 0.7 |
| Punica_granatum_1 | nad5 | 1918 | 640 | 644 | CGT (R) => TGT (C) | 0.7 |
| Punica_granatum_1 | nad5 | 1958 | 653 | 657 | TCG (S) => TTG (L) | 0.9 |
| Punica_granatum_1 | nad9 | 92 | 31 | 31 | TCT (S) => TTT (F) | 0.75 |
| Punica_granatum_1 | nad9 | 113 | 38 | 38 | CCA (P) => CTA (L) | 0.92 |
| Punica_granatum_1 | nad9 | 167 | 56 | 56 | TCG (S) => TTG (L) | 0.92 |
| Punica_granatum_1 | nad9 | 190 | 64 | 64 | CAT (H) => TAT (Y) | 1 |
| Punica_granatum_1 | nad9 | 328 | 110 | 110 | CGG (R) => TGG (W) | 1 |
| Punica_granatum_1 | nad9 | 368 | 123 | 123 | TCC (S) => TTC (F) | 1 |
| Punica_granatum_1 | nad9 | 398 | 133 | 133 | TCA (S) => TTA (L) | 1 |
| Punica_granatum_1 | nad9 | 439 | 147 | 147 | CTT (L) => TTT (F) | 1 |
| Punica_granatum_1 | rpl5 | 35 | 12 | 17 | TCA (S) => TTA (L) | 0.78 |
| Punica_granatum_1 | rpl5 | 59 | 20 | 25 | CCG (P) => CTG (L) | 0.89 |
| Punica_granatum_1 | rpl5 | 64 | 22 | 27 | CAC (H) => TAC (Y) | 1 |
| Punica_granatum_1 | rpl5 | 92 | 31 | 36 | TCG (S) => TTG (L) | 1 |
| Punica_granatum_1 | rpl5 | 175 | 59 | 64 | CGC (R) => TGC (C) | 0.89 |
| Punica_granatum_1 | rpl5 | 335 | 112 | 125 | TCG (S) => TTG (L) | 0.44 |
| Punica_granatum_1 | rpl5 | 518 | 173 | 188 | CCA (P) => CTA (L) | 0.89 |
| Punica_granatum_1 | rps12 | 284 | 95 | 95 | TCC (S) => TTC (F) | 0.76 |
| Punica_granatum_1 | rps14 | 47 | 16 | 16 | GCG (A) => GTG (V) | 0.6 |
| Punica_granatum_1 | rps14 | 194 | 65 | 65 | TCC (S) => TTC (F) | 0.6 |
| Punica_granatum_1 | rps14 | 271 | 91 | 91 | CCT (P) => TCT (S) | 0.6 |
| Punica_granatum_1 | sdh4 | 155 | 52 | 52 | CCA (P) => CTA (L) | 0.88 |
| Punica_granatum_1 | sdh4 | 203 | 68 | 68 | TCA (S) => TTA (L) | 0.75 |
| Punica_granatum_1 | sdh4 | 259 | 87 | 87 | CAT (H) => TAT (Y) | 0.88 |
| Punica_granatum_1 | sdh4 | 353 | 118 | 118 | TCT (S) => TTT (F) | 0.5 |
| Punica_granatum_2 | atp6 | 37 | 13 | 30 | CCA (P) => TCA (S) | 0.75 |
| Punica_granatum_2 | atp6 | 116 | 39 | 56 | TCA (S) => TTA (L) | 1 |
| Punica_granatum_2 | atp6 | 173 | 58 | 75 | CCG (P) => CTG (L) | 1 |
| Punica_granatum_2 | atp6 | 229 | 77 | 94 | CGC (R) => TGC (C) | 0.75 |
| Punica_granatum_2 | atp6 | 236 | 79 | 96 | TCG (S) => TTG (L) | 0.67 |
| Punica_granatum_2 | atp6 | 254 | 85 | 102 | TCG (S) => TTG (L) | 1 |
| Punica_granatum_2 | atp6 | 262 | 88 | 105 | CGT (R) => TGT (C) | 1 |
| Punica_granatum_2 | atp6 | 269 | 90 | 107 | CCC (P) => CTC (L) | 1 |
| Punica_granatum_2 | atp6 | 401 | 134 | 151 | TCA (S) => TTA (L) | 1 |
| Punica_granatum_2 | atp6 | 460 | 154 | 171 | CCT (P) => TCT (S) | 1 |
| Punica_granatum_2 | atp6 | 463 | 155 | 172 | CAT (H) => TAT (Y) | 1 |
| Punica_granatum_2 | atp6 | 485 | 162 | 179 | TCA (S) => TTA (L) | 1 |
| Punica_granatum_2 | atp6 | 527 | 176 | 193 | TCA (S) => TTA (L) | 1 |
| Punica_granatum_2 | atp6 | 548 | 183 | 200 | TCC (S) => TTC (F) | 1 |
| Punica_granatum_2 | atp6 | 664 | 222 | 239 | CAT (H) => TAT (Y) | 1 |
| Punica_granatum_2 | atp6 | 671 | 224 | 241 | TCT (S) => TTT (F) | 1 |
| Punica_granatum_2 | atp6 | 680 | 227 | 244 | TCA (S) => TTA (L) | 1 |
| Punica_granatum_2 | atp6 | 707 | 236 | 253 | ACA (T) => ATA (I) | 0.92 |
| Punica_granatum_2 | atp6 | 718 | 240 | 257 | CAA (Q) => TAA (X) | 1 |
| Punica_granatum_2 | ccmB | 28 | 10 | 10 | CAT (H) => TAT (Y) | 0.89 |
| Punica_granatum_2 | ccmB | 43 | 15 | 15 | CCC (P) => TCC (S) | 0.67 |
| Punica_granatum_2 | ccmB | 71 | 24 | 26 | CCG (P) => CTG (L) | 1 |
| Punica_granatum_2 | ccmB | 80 | 27 | 29 | TCG (S) => TTG (L) | 1 |
| Punica_granatum_2 | ccmB | 128 | 43 | 45 | TCA (S) => TTA (L) | 1 |
| Punica_granatum_2 | ccmB | 137 | 46 | 48 | TCC (S) => TTC (F) | 1 |
| Punica_granatum_2 | ccmB | 149 | 50 | 52 | CCG (P) => CTG (L) | 1 |
| Punica_granatum_2 | ccmB | 160 | 54 | 56 | CCT (P) => TCT (S) | 0.67 |
| Punica_granatum_2 | ccmB | 164 | 55 | 57 | CCG (P) => CTG (L) | 0.89 |
| Punica_granatum_2 | ccmB | 172 | 58 | 60 | CCT (P) => TCT (S) | 0.89 |
| Punica_granatum_2 | ccmB | 179 | 60 | 62 | CCT (P) => CTT (L) | 1 |
| Punica_granatum_2 | ccmB | 181 | 61 | 63 | CCC (P) => TCC (S) | 0.78 |
| Punica_granatum_2 | ccmB | 193 | 65 | 69 | CCT (P) => TTT (F) | 0.89 |
| Punica_granatum_2 | ccmB | 194 | 65 | 69 | CCT (P) => TTT (F) | 0.89 |
| Punica_granatum_2 | ccmB | 286 | 96 | 102 | CGG (R) => TGG (W) | 1 |
| Punica_granatum_2 | ccmB | 304 | 102 | 108 | CGT (R) => TGT (C) | 0.78 |
| Punica_granatum_2 | ccmB | 313 | 105 | 111 | CGT (R) => TGT (C) | 0.89 |
| Punica_granatum_2 | ccmB | 367 | 123 | 129 | CGG (R) => TGG (W) | 0.78 |
| Punica_granatum_2 | ccmB | 392 | 131 | 137 | CCG (P) => CTG (L) | 0.89 |
| Punica_granatum_2 | ccmB | 424 | 142 | 148 | CGT (R) => TGT (C) | 0.89 |
| Punica_granatum_2 | ccmB | 428 | 143 | 149 | TCG (S) => TTG (L) | 1 |
| Punica_granatum_2 | ccmB | 467 | 156 | 164 | TCG (S) => TTG (L) | 0.89 |
| Punica_granatum_2 | ccmB | 476 | 159 | 167 | CCA (P) => CTA (L) | 0.89 |
| Punica_granatum_2 | ccmB | 485 | 162 | 170 | TCA (S) => TTA (L) | 1 |
| Punica_granatum_2 | ccmB | 494 | 165 | 173 | TCA (S) => TTA (L) | 1 |
| Punica_granatum_2 | ccmB | 503 | 168 | 176 | CCA (P) => CTA (L) | 1 |
| Punica_granatum_2 | ccmB | 512 | 171 | 179 | TCT (S) => TTT (F) | 1 |
| Punica_granatum_2 | ccmB | 514 | 172 | 180 | CGT (R) => TGT (C) | 1 |
| Punica_granatum_2 | ccmB | 548 | 183 | 191 | CCT (P) => CTT (L) | 0.78 |
| Punica_granatum_2 | ccmB | 551 | 184 | 192 | TCA (S) => TTA (L) | 1 |
| Punica_granatum_2 | ccmB | 554 | 185 | 193 | TCG (S) => TTG (L) | 0.89 |
| Punica_granatum_2 | ccmB | 566 | 189 | 197 | TCC (S) => TTC (F) | 0.78 |
| Punica_granatum_2 | ccmB | 569 | 190 | 198 | TCT (S) => TTT (F) | 0.78 |
| Punica_granatum_2 | ccmB | 572 | 191 | 199 | CCG (P) => CTG (L) | 1 |
| Punica_granatum_2 | ccmB | 596 | 199 | 207 | TCG (S) => TTG (L) | 0.89 |
| Punica_granatum_2 | ccmB | 611 | 204 | 212 | TCA (S) => TTA (L) | 0.89 |
| Punica_granatum_2 | ccmFc | 38 | 13 | 13 | TCC (S) => TTC (F) | 0.83 |
| Punica_granatum_2 | ccmFc | 50 | 17 | 17 | CCT (P) => CTT (L) | 1 |
| Punica_granatum_2 | ccmFc | 52 | 18 | 18 | CGT (R) => TGT (C) | 1 |
| Punica_granatum_2 | ccmFc | 103 | 35 | 35 | CCC (P) => TCC (S) | 1 |
| Punica_granatum_2 | ccmFc | 119 | 40 | 40 | TCT (S) => TTT (F) | 1 |
| Punica_granatum_2 | ccmFc | 122 | 41 | 41 | TCC (S) => TTC (F) | 1 |
| Punica_granatum_2 | ccmFc | 146 | 49 | 49 | CCT (P) => CTT (L) | 1 |
| Punica_granatum_2 | ccmFc | 151 | 51 | 51 | CCT (P) => TCT (S) | 0.83 |
| Punica_granatum_2 | ccmFc | 155 | 52 | 52 | TCA (S) => TTA (L) | 1 |
| Punica_granatum_2 | ccmFc | 160 | 54 | 54 | CCT (P) => TCT (S) | 0.67 |
| Punica_granatum_2 | ccmFc | 305 | 102 | 102 | TCA (S) => TTA (L) | 0.83 |
| Punica_granatum_2 | ccmFc | 310 | 104 | 104 | CGT (R) => TGT (C) | 0.5 |
| Punica_granatum_2 | ccmFc | 391 | 131 | 131 | CGT (R) => TGT (C) | 1 |
| Punica_granatum_2 | ccmFc | 406 | 136 | 136 | CGT (R) => TGT (C) | 0.83 |
| Punica_granatum_2 | ccmFc | 412 | 138 | 138 | CTC (L) => TTC (F) | 0.67 |
| Punica_granatum_2 | ccmFc | 704 | 235 | 239 | GCT (A) => GTT (V) | 0.83 |
| Punica_granatum_2 | ccmFc | 884 | 295 | 299 | TCT (S) => TTT (F) | 0.83 |
| Punica_granatum_2 | ccmFc | 1148 | 383 | 390 | CCA (P) => CTA (L) | 1 |
| Punica_granatum_2 | ccmFc | 1169 | 390 | 397 | TCG (S) => TTG (L) | 1 |
| Punica_granatum_2 | ccmFc | 1243 | 415 | 422 | CGG (R) => TGG (W) | 1 |
| Punica_granatum_2 | ccmFc | 1324 | 442 | 449 | CGA (R) => TGA (X) | 1 |
| Punica_granatum_2 | matR | 32 | 11 | 31 | TCC (S) => TTC (F) | 0.62 |
| Punica_granatum_2 | matR | 193 | 65 | 85 | CCA (P) => TCA (S) | 0.75 |
| Punica_granatum_2 | matR | 236 | 79 | 99 | TCC (S) => TTC (F) | 0.62 |
| Punica_granatum_2 | matR | 292 | 98 | 118 | CCC (P) => TCC (S) | 1 |
| Punica_granatum_2 | matR | 923 | 308 | 333 | TCA (S) => TTA (L) | 1 |
| Punica_granatum_2 | matR | 1679 | 560 | 586 | TCC (S) => TTC (F) | 1 |
| Punica_granatum_2 | matR | 1700 | 567 | 593 | CCT (P) => CTT (L) | 1 |
| Punica_granatum_2 | matR | 1756 | 586 | 612 | CAC (H) => TAC (Y) | 1 |
| Punica_granatum_2 | matR | 1844 | 615 | 641 | TCA (S) => TTA (L) | 0.88 |
| Punica_granatum_2 | rpl10 | 101 | 34 | 34 | TCG (S) => TTG (L) | 0.83 |
| Punica_granatum_2 | rpl10 | 134 | 45 | 45 | CCA (P) => CTA (L) | 0.83 |
| Punica_granatum_2 | rpl10 | 239 | 80 | 80 | TCG (S) => TTG (L) | 0.83 |
| Punica_granatum_2 | rpl10 | 314 | 105 | 105 | TCA (S) => TTA (L) | 0.83 |
| Punica_granatum_2 | rpl16 | 14 | 5 | 108 | TCC (S) => TTC (F) | 0.89 |
| Punica_granatum_2 | rpl16 | 46 | 16 | 122 | CTC (L) => TTC (F) | 0.89 |
| Punica_granatum_2 | rpl16 | 215 | 72 | 178 | CCA (P) => CTA (L) | 1 |
| Punica_granatum_2 | rpl16 | 221 | 74 | 180 | TCG (S) => TTG (L) | 0.75 |
| Punica_granatum_2 | rps19 | 137 | 46 | 67 | TCC (S) => TTC (F) | 0.33 |
| Punica_granatum_2 | rps19 | 163 | 55 | 76 | CCT (P) => TTT (F) | 1 |
| Punica_granatum_2 | rps19 | 164 | 55 | 76 | CCT (P) => TTT (F) | 1 |
| Punica_granatum_2 | rps3 | 92 | 31 | 31 | TCA (S) => TTA (L) | 0.71 |
| Punica_granatum_2 | rps3 | 518 | 173 | 178 | TCA (S) => TTA (L) | 0.71 |
| Punica_granatum_2 | rps3 | 631 | 211 | 225 | CTT (L) => TTT (F) | 0.83 |
| Punica_granatum_2 | rps3 | 707 | 236 | 250 | CCC (P) => CTC (L) | 0.71 |
| Punica_granatum_2 | rps3 | 737 | 246 | 260 | TCG (S) => TTG (L) | 0.86 |
| Punica_granatum_2 | rps3 | 1388 | 463 | 489 | CCG (P) => CTG (L) | 1 |
| Punica_granatum_2 | rps3 | 1415 | 472 | 498 | CCG (P) => CTG (L) | 0.86 |
| Punica_granatum_2 | rps3 | 1526 | 509 | 535 | TCA (S) => TTA (L) | 1 |
| Punica_granatum_2 | rps3 | 1579 | 527 | 554 | CGT (R) => TGT (C) | 1 |
| Punica_granatum_2 | rps3 | 1612 | 538 | 565 | CCT (P) => TCT (S) | 0.57 |
| Punica_granatum_2 | rps3 | 1672 | 558 | 587 | CAA (Q) => TAA (X) | 0.29 |
| Punica_granatum_3 | cox1 | 32 | 11 | 185 | TCA (S) => TTA (L) | 1 |
| Punica_granatum_3 | cox1 | 56 | 19 | 193 | CCA (P) => CTA (L) | 1 |
| Punica_granatum_3 | cox1 | 71 | 24 | 198 | CCA (P) => CTA (L) | 1 |
| Punica_granatum_3 | cox1 | 149 | 50 | 224 | TCT (S) => TTT (F) | 1 |
| Punica_granatum_3 | cox1 | 196 | 66 | 240 | CGG (R) => TGG (W) | 1 |
| Punica_granatum_3 | cox1 | 227 | 76 | 250 | CCC (P) => CTC (L) | 1 |
| Punica_granatum_3 | cox1 | 242 | 81 | 255 | TCC (S) => TTC (F) | 1 |
| Punica_granatum_3 | cox1 | 518 | 173 | 347 | TCC (S) => TTC (F) | 1 |
| Punica_granatum_3 | cox1 | 560 | 187 | 361 | CCG (P) => CTG (L) | 1 |
| Punica_granatum_3 | cox1 | 667 | 223 | 397 | CAC (H) => TAC (Y) | 1 |
| Punica_granatum_3 | cox1 | 755 | 252 | 426 | CCG (P) => CTG (L) | 0.89 |
| Punica_granatum_3 | cox1 | 760 | 254 | 428 | CTC (L) => TTC (F) | 1 |
| Punica_granatum_3 | cox1 | 883 | 295 | 469 | CGT (R) => TGT (C) | 0.67 |
| Punica_granatum_3 | cox1 | 914 | 305 | 479 | TCA (S) => TTA (L) | 1 |
| Punica_granatum_3 | cox2 | 71 | 24 | 26 | TCT (S) => TTT (F) | 1 |
| Punica_granatum_3 | cox2 | 161 | 54 | 56 | TCA (S) => TTA (L) | 0.95 |
| Punica_granatum_3 | cox2 | 163 | 55 | 57 | CGG (R) => TGG (W) | 1 |
| Punica_granatum_3 | cox2 | 253 | 85 | 87 | CGG (R) => TGG (W) | 1 |
| Punica_granatum_3 | cox2 | 263 | 88 | 90 | TCT (S) => TTT (F) | 0.95 |
| Punica_granatum_3 | cox2 | 278 | 93 | 95 | CCG (P) => CTG (L) | 1 |
| Punica_granatum_3 | cox2 | 379 | 127 | 129 | CGG (R) => TGG (W) | 1 |
| Punica_granatum_3 | cox2 | 443 | 148 | 150 | ACG (T) => ATG (M) | 1 |
| Punica_granatum_3 | cox2 | 476 | 159 | 161 | TCA (S) => TTA (L) | 1 |
| Punica_granatum_3 | cox2 | 544 | 182 | 184 | CCT (P) => TCT (S) | 1 |
| Punica_granatum_3 | cox2 | 557 | 186 | 188 | CCT (P) => CTT (L) | 1 |
| Punica_granatum_3 | cox2 | 581 | 194 | 196 | TCA (S) => TTA (L) | 1 |
| Punica_granatum_3 | cox2 | 632 | 211 | 213 | TCG (S) => TTG (L) | 0.84 |
| Punica_granatum_3 | cox2 | 698 | 233 | 235 | ACG (T) => ATG (M) | 1 |
| Punica_granatum_3 | cox2 | 721 | 241 | 243 | CCT (P) => TCT (S) | 0.79 |
| Punica_granatum_3 | cox2 | 742 | 248 | 250 | CGG (R) => TGG (W) | 1 |
| Punica_granatum_3 | mttB | 56 | 19 | 164 | TCG (S) => TTG (L) | 0.88 |
| Punica_granatum_3 | mttB | 64 | 22 | 167 | CCC (P) => TCC (S) | 1 |
| Punica_granatum_3 | mttB | 107 | 36 | 181 | CCA (P) => CTA (L) | 0.62 |
| Punica_granatum_3 | mttB | 113 | 38 | 183 | CCT (P) => CTT (L) | 0.5 |
| Punica_granatum_3 | mttB | 169 | 57 | 202 | CCG (P) => TCG (S) | 1 |
| Punica_granatum_3 | mttB | 226 | 76 | 221 | CGT (R) => TGT (C) | 0.62 |
| Punica_granatum_3 | mttB | 272 | 91 | 236 | TCG (S) => TTG (L) | 0.75 |
| Punica_granatum_3 | mttB | 329 | 110 | 255 | ACT (T) => ATT (I) | 0.67 |
| Punica_granatum_3 | nad7 | 38 | 13 | 13 | TCG (S) => TTG (L) | 0.75 |
| Punica_granatum_3 | nad7 | 77 | 26 | 26 | TCA (S) => TTA (L) | 1 |
| Punica_granatum_3 | nad7 | 83 | 28 | 28 | TCA (S) => TTA (L) | 1 |
| Punica_granatum_3 | nad7 | 200 | 67 | 67 | TCT (S) => TTT (F) | 1 |
| Punica_granatum_3 | nad7 | 224 | 75 | 75 | ACG (T) => ATG (M) | 1 |
| Punica_granatum_3 | nad7 | 244 | 82 | 82 | CAT (H) => TAT (Y) | 1 |
| Punica_granatum_3 | nad7 | 251 | 84 | 84 | TCA (S) => TTA (L) | 1 |
| Punica_granatum_3 | nad7 | 316 | 106 | 106 | CGT (R) => TGT (C) | 1 |
| Punica_granatum_3 | nad7 | 335 | 112 | 112 | TCA (S) => TTA (L) | 0.88 |
| Punica_granatum_3 | nad7 | 344 | 115 | 115 | TCA (S) => TTA (L) | 1 |
| Punica_granatum_3 | nad7 | 383 | 128 | 128 | TCA (S) => TTA (L) | 1 |
| Punica_granatum_3 | nad7 | 533 | 178 | 178 | TCC (S) => TTC (F) | 1 |
| Punica_granatum_3 | nad7 | 578 | 193 | 193 | TCA (S) => TTA (L) | 0.88 |
| Punica_granatum_3 | nad7 | 679 | 227 | 227 | CCA (P) => TCA (S) | 0.88 |
| Punica_granatum_3 | nad7 | 698 | 233 | 233 | TCG (S) => TTG (L) | 1 |
| Punica_granatum_3 | nad7 | 724 | 242 | 242 | CAT (H) => TAT (Y) | 1 |
| Punica_granatum_3 | nad7 | 734 | 245 | 245 | TCG (S) => TTG (L) | 0.88 |
| Punica_granatum_3 | nad7 | 739 | 247 | 247 | CTT (L) => TTT (F) | 1 |
| Punica_granatum_3 | nad7 | 769 | 257 | 257 | CGC (R) => TGC (C) | 1 |
| Punica_granatum_3 | nad7 | 926 | 309 | 310 | TCA (S) => TTA (L) | 0.88 |
| Punica_granatum_3 | nad7 | 973 | 325 | 326 | CCT (P) => TCT (S) | 0.88 |
| Punica_granatum_3 | nad7 | 1057 | 353 | 354 | CGT (R) => TGT (C) | 1 |
| Punica_granatum_3 | nad7 | 1079 | 360 | 361 | TCT (S) => TTT (F) | 1 |
| Punica_granatum_3 | nad7 | 1088 | 363 | 364 | TCA (S) => TTA (L) | 1 |
| Punica_granatum_3 | nad7 | 1103 | 368 | 369 | TCT (S) => TTT (F) | 1 |
| Punica_granatum_3 | nad7 | 1124 | 375 | 376 | CCA (P) => CTA (L) | 1 |
| Punica_granatum_3 | nad7 | 1166 | 389 | 390 | TCT (S) => TTT (F) | 1 |
| Punica_granatum_3 | rps1 | 125 | 42 | 59 | CCT (P) => CTT (L) | 0.67 |
| Punica_granatum_3 | rps1 | 482 | 161 | 178 | TCA (S) => TTA (L) | 0.67 |
| Punica_granatum_4 | nad6 | 7 | 3 | 5 | CTT (L) => TTT (F) | 1 |
| Punica_granatum_4 | nad6 | 26 | 9 | 11 | CCT (P) => CTT (L) | 0.4 |
| Punica_granatum_4 | nad6 | 53 | 18 | 20 | GCA (A) => GTA (V) | 0.7 |
| Punica_granatum_4 | nad6 | 88 | 30 | 32 | CCC (P) => TTC (F) | 0.7 |
| Punica_granatum_4 | nad6 | 89 | 30 | 32 | CCC (P) => TTC (F) | 0.7 |
| Punica_granatum_4 | nad6 | 95 | 32 | 34 | CCA (P) => CTA (L) | 1 |
| Punica_granatum_4 | nad6 | 103 | 35 | 37 | CGC (R) => TGC (C) | 1 |
| Punica_granatum_4 | nad6 | 146 | 49 | 51 | TCC (S) => TTC (F) | 1 |
| Punica_granatum_4 | nad6 | 161 | 54 | 56 | CCA (P) => CTA (L) | 1 |
| Punica_granatum_4 | nad6 | 191 | 64 | 66 | TCA (S) => TTA (L) | 1 |
| Punica_granatum_4 | nad6 | 289 | 97 | 99 | CTT (L) => TTT (F) | 1 |
| Punica_granatum_4 | nad6 | 446 | 149 | 151 | TCC (S) => TTC (F) | 1 |
| Punica_granatum_4 | rpl2 | 404 | 135 | 138 | GCG (A) => GTG (V) | 1 |
| Punica_granatum_4 | rpl2 | 485 | 162 | 167 | GCG (A) => GTG (V) | 0.67 |
| Punica_granatum_4 | rps4 | 38 | 13 | 26 | TCA (S) => TTA (L) | 0.5 |
| Punica_granatum_4 | rps4 | 49 | 17 | 30 | CGG (R) => TGG (W) | 1 |
| Punica_granatum_4 | rps4 | 482 | 161 | 175 | TCA (S) => TTA (L) | 1 |
| Punica_granatum_4 | rps4 | 529 | 177 | 205 | CGG (R) => TGG (W) | 0.83 |
| Punica_granatum_4 | rps4 | 914 | 305 | 349 | TCG (S) => TTG (L) | 0.83 |
| Punica_granatum_4 | rps4 | 925 | 309 | 353 | CAT (H) => TAT (Y) | 0.83 |
| Punica_granatum_4 | rps4 | 935 | 312 | 356 | CCA (P) => CTA (L) | 0.67 |
| Punica_granatum_4 | rps4 | 950 | 317 | 361 | TCT (S) => TTT (F) | 1 |
| Punica_granatum_4 | rps4 | 1001 | 334 | 378 | CCA (P) => CTA (L) | 0.83 |
| Punica_granatum_4 | rps4 | 1010 | 337 | 381 | CCT (P) => CTT (L) | 1 |
| Punica_granatum_4 | rps4 | 1015 | 339 | 383 | CGG (R) => TGG (W) | 1 |
| Punica_granatum_5 | rps7 | 116 | 39 | 39 | CCA (P) => CTA (L) | 0.75 |
| Punica_granatum_5 | rps7 | 332 | 111 | 134 | TCA (S) => TTA (L) | 0.88 |
| Punica_granatum_5 | rps7 | 335 | 112 | 135 | TCT (S) => TTT (F) | 0.62 |

Note: ID: sequence name; Gene: The gene where RNA editing occurs; Nt pos: Position of RNA editing in CDS; AA Pos: amino acid location where RNA editing occurs; Align Col: the aligned position where RNA editing occurs; Effect: The effect of RNA editing; Score: The score of the site (0-1)

**Table S5.** **The nucleotide variability of *P. granatum* mitogenome**

| Number | Region | Pi | Total Number of mutations | Region length |
| --- | --- | --- | --- | --- |
| 1 | gene1.cob | 0.01768 | 37 | 1890 |
| 2 | gene10.nad9 | 0.10582 | 116 | 608 |
| 3 | gene11.rps12 | 0.01279 | 9 | 378 |
| 4 | gene12.nad3 | 0.03595 | 24 | 357 |
| 5 | gene13.ccmFn | 0.03422 | 89 | 1743 |
| 6 | gene14.nad4 | 0.0186 | 51 | 1521 |
| 7 | gene15.atp1 | 0.04771 | 128 | 1536 |
| 8 | gene16.rrn26 | 0.00962 | 47 | 3632 |
| 9 | gene17.rpl16 | 0.02142 | 8 | 327 |
| 10 | gene18.rps3 | 0.05392 | 160 | 1740 |
| 11 | gene19.rps19 | 0 | 0 | 285 |
| 12 | gene2.rps14 | 0.00715 | 4 | 303 |
| 13 | gene20.nad1 | 0.01285 | 21 | 1956 |
| 14 | gene21.atp6 | 0.03107 | 38 | 1240 |
| 15 | gene22.matR | 0.03799 | 81 | 2041 |
| 16 | gene23.ccmFc | 0.0271 | 67 | 1374 |
| 17 | gene24.ccmB | 0.02469 | 30 | 624 |
| 18 | gene25.rpl10 | 0.0426 | 40 | 531 |
| 19 | gene26.cox2 | 0.04573 | 56 | 838 |
| 20 | gene27.rps1 | 0.05243 | 45 | 681 |
| 21 | gene28.nad7 | 0.00893 | 19 | 1350 |
| 22 | gene29.cox1 | 0.02066 | 41 | 1584 |
| 23 | gene3.rpl5 | 0.02453 | 25 | 564 |
| 24 | gene30.rpl2 | 0.03798 | 61 | 1074 |
| 25 | gene31.rps4 | 0.03908 | 70 | 1182 |
| 26 | gene32.nad6 | 0.02805 | 32 | 618 |
| 27 | gene33.rrn18 | 0.0698 | 205 | 2706 |
| 28 | gene34.rrn5 | 0.00862 | 2 | 121 |
| 29 | gene35.nad2 | 0.01262 | 32 | 3087 |
| 30 | gene36.nad5 | 0.01208 | 43 | 6039 |
| 31 | gene37.atp9 | 0.07259 | 30 | 225 |
| 32 | gene38.mttB | 0.04971 | 25 | 798 |
| 33 | gene39.rps7 | 0.02424 | 20 | 450 |
| 34 | gene4.ccmC | 0.02707 | 34 | 770 |
| 35 | gene5.atp8 | 0.04485 | 21 | 480 |
| 36 | gene6.cox3 | 0.01713 | 24 | 798 |
| 37 | gene7.sdh4 | 0.02929 | 17 | 576 |
| 38 | gene8.atp4 | 0.04167 | 42 | 597 |
| 39 | gene9.nad4L | 0.01343 | 7 | 303 |

**Table S6. Comparison information of chloroplast and mitochondrial genome in *P. granatum***

| query-chl | subject-mt | percentage of identical matches | length | number of mismatches | number of gap openings | start of alignment in query | end of alignment in query | start of alignment in subject | end of alignment in subject | expect value | bitscore | gene |
| --- | --- | --- | --- | --- | --- | --- | --- | --- | --- | --- | --- | --- |
| Chloroplast | chr7 | 100 | 3726 | 0 | 0 | 139435 | 143160 | 3726 | 1 | 0 | 6881 | trnA-UGC (partical:63.01%); trnI-GAU; rrn16; trnV-GAC |
| Chloroplast | chr7 | 100 | 3726 | 0 | 0 | 104500 | 108225 | 1 | 3726 | 0 | 6881 | trnV-GAC; rrn16; trnI-GAU; trnA-UGC (partical:63.01%) |
| Chloroplast | chr5 | 100 | 2586 | 0 | 0 | 108226 | 110811 | 24105 | 21520 | 0 | 4776 | trnA-UGC (partical:36.99%); rrn23(partical:75.09%) |
| Chloroplast | chr5 | 100 | 2586 | 0 | 0 | 136849 | 139434 | 21520 | 24105 | 0 | 4776 | rrn2 3(partical:75.09%); trnA-UGC (partical:36.99%) |
| Chloroplast | chr5 | 100 | 28 | 0 | 0 | 44067 | 44094 | 15025 | 14998 | 7.60E-06 | 52.8 | psaA (partical:1.24%) |
| Chloroplast | chr2 | 100 | 1165 | 0 | 0 | 143161 | 144325 | 93198 | 92034 | 0 | 2152 |  |
| Chloroplast | chr2 | 100 | 1165 | 0 | 0 | 103335 | 104499 | 92034 | 93198 | 0 | 2152 |  |
| Chloroplast | chr2 | 92.039 | 716 | 48 | 7 | 37593 | 38303 | 42614 | 41903 | 0 | 998 | psbC (partical:26.44%); trnS-UGA |
| Chloroplast | chr2 | 95.332 | 557 | 10 | 7 | 111828 | 112372 | 87404 | 86852 | 0 | 870 | rrn5; trnR-ACG(partical:79.73%) |
| Chloroplast | chr2 | 95.332 | 557 | 10 | 7 | 135288 | 135832 | 86852 | 87404 | 0 | 870 | trnR-ACG (partical:79.73%); rrn5 |
| Chloroplast | chr2 | 96.471 | 85 | 2 | 1 | 20 | 104 | 84703 | 84786 | 5.67E-32 | 139 | trnH-GUG |
| Chloroplast | chr2 | 92.405 | 79 | 5 | 1 | 56030 | 56108 | 81377 | 81300 | 1.24E-23 | 111 | trnM-CAU |
| Chloroplast | chr4 | 74.157 | 890 | 173 | 42 | 141780 | 142643 | 21693 | 20835 | 2.44E-85 | 316 | rrn16 (partical:57.95%) |
| Chloroplast | chr4 | 74.157 | 890 | 173 | 42 | 105017 | 105880 | 20835 | 21693 | 2.44E-85 | 316 | rrn16 (partical:57.95%) |
| Chloroplast | chr1 | 93.711 | 159 | 9 | 1 | 70783 | 70941 | 47368 | 47525 | 1.95E-61 | 237 | trnW-CCA |
| Chloroplast | chr1 | 82.456 | 285 | 29 | 11 | 32154 | 32422 | 72980 | 73259 | 3.27E-59 | 230 | trnD-GUC |
| Chloroplast | chr1 | 91.558 | 154 | 13 | 0 | 71018 | 71171 | 47579 | 47732 | 3.29E-54 | 213 | trnP-UGG |
| Chloroplast | chr3 | 83.902 | 205 | 33 | 0 | 131048 | 131252 | 48980 | 48776 | 3.32E-49 | 196 | ycf1 (partical:3.64%) |
| Chloroplast | chr3 | 97.619 | 84 | 2 | 0 | 113010 | 113093 | 13844 | 13761 | 1.22E-33 | 145 | trnN-GUU |
| Chloroplast | chr3 | 97.619 | 84 | 2 | 0 | 134567 | 134650 | 13761 | 13844 | 1.22E-33 | 145 | trnN-GUU |
| Chloroplast | chr6 | 93.827 | 81 | 4 | 1 | 114211 | 114290 | 3393 | 3313 | 2.05E-26 | 121 | ycf1 (partical:7.37%) |
| Chloroplast | chr6 | 93.827 | 81 | 4 | 1 | 133370 | 133449 | 3313 | 3393 | 2.05E-26 | 121 | ycf1 (partical:1.42%) |

| query-chl-pep | subject-mt | percentage of identical matches | length | number of mismatches | number of gap openings | start of alignment in query | end of alignment in query | start of alignment in subject | end of alignment in subject | expect value | bitscore |
| --- | --- | --- | --- | --- | --- | --- | --- | --- | --- | --- | --- |
| atpA_len507 | chr1 | 61.783 | 471 | 171 | 1 | 5 | 466 | 101189 | 99777 | 3.14E-180 | 556 |
| atpB_len498 | chr1 | 25 | 384 | 257 | 9 | 69 | 436 | 101003 | 99897 | 2.24E-20 | 89.4 |
| ndhA_len363 | chr1 | 62.857 | 35 | 13 | 0 | 215 | 249 | 25059 | 25163 | 3.44E-08 | 49.3 |
| ndhA_len363 | chr2 | 43.689 | 103 | 58 | 0 | 51 | 153 | 43498 | 43806 | 7.75E-21 | 88.6 |
| ndhB_len510 | chr1 | 31.481 | 108 | 74 | 0 | 91 | 198 | 106191 | 105868 | 5.75E-11 | 59.3 |
| ndhB_len510 | chr3 | 34.956 | 226 | 133 | 5 | 246 | 462 | 17160 | 17822 | 1.61E-11 | 60.8 |
| ndhB_len510 | chr4 | 31.481 | 108 | 74 | 0 | 91 | 198 | 36877 | 36554 | 5.71E-11 | 59.3 |
| ndhC_len120 | chr1 | 39.474 | 76 | 39 | 2 | 13 | 84 | 55602 | 55384 | 9.10E-14 | 60.5 |
| ndhD_len500 | chr1 | 26.667 | 180 | 122 | 4 | 224 | 400 | 116819 | 116301 | 2.59E-06 | 43.9 |
| ndhD_len500 | chr1 | 29.06 | 117 | 76 | 1 | 327 | 443 | 82456 | 82785 | 1.69E-09 | 54.3 |
| ndhD_len500 | chr1 | 33.962 | 159 | 101 | 2 | 150 | 307 | 77891 | 78358 | 3.89E-20 | 88.6 |
| ndhD_len500 | chr4 | 26.667 | 180 | 122 | 4 | 224 | 400 | 47505 | 46987 | 2.58E-06 | 43.9 |
| ndhF_len749 | chr1 | 46.742 | 353 | 171 | 5 | 110 | 454 | 117218 | 116187 | 1.11E-71 | 250 |
| ndhF_len749 | chr3 | 31.818 | 176 | 105 | 6 | 246 | 417 | 17193 | 17687 | 1.91E-12 | 65.1 |
| ndhF_len749 | chr4 | 46.742 | 353 | 171 | 5 | 110 | 454 | 47904 | 46873 | 1.09E-71 | 250 |
| ndhH_len393 | chr3 | 38.312 | 154 | 95 | 0 | 72 | 225 | 53455 | 53916 | 1.23E-26 | 106 |
| ndhH_len393 | chr3 | 41.176 | 51 | 28 | 1 | 214 | 264 | 20443 | 20589 | 5.88E-06 | 42.4 |
| ndhH_len393 | chr3 | 42.222 | 45 | 26 | 0 | 14 | 58 | 51048 | 51182 | 4.51E-06 | 42.7 |
| ndhH_len393 | chr3 | 48 | 75 | 39 | 0 | 319 | 393 | 56965 | 57189 | 8.12E-18 | 79.7 |
| ndhJ_len158 | chr1 | 41.667 | 60 | 35 | 0 | 96 | 155 | 48655 | 48476 | 8.17E-08 | 44.7 |
| petB_len215 | chr1 | 34.921 | 189 | 122 | 1 | 27 | 215 | 1972 | 1409 | 2.38E-36 | 129 |
| petD_len160 | chr1 | 32.692 | 104 | 61 | 3 | 38 | 133 | 1369 | 1061 | 8.15E-12 | 56.6 |
| psbC_len473 | chr2 | 90.909 | 132 | 9 | 1 | 340 | 471 | 42634 | 42248 | 1.25E-52 | 186 |
| rpl16_len135 | chr2 | 43.564 | 101 | 49 | 1 | 18 | 110 | 34953 | 34651 | 3.27E-23 | 88.2 |
| rpl2_len274 | chr4 | 44.776 | 134 | 68 | 2 | 107 | 235 | 9435 | 9037 | 8.32E-24 | 95.1 |
| rpl2_len274 | chr4 | 48.333 | 60 | 30 | 1 | 38 | 97 | 11964 | 11788 | 7.05E-12 | 59.7 |
| rps12_len123 | chr1 | 62.602 | 123 | 46 | 0 | 1 | 123 | 55224 | 54856 | 3.40E-49 | 161 |
| rps14_len100 | chr1 | 40 | 80 | 46 | 1 | 23 | 100 | 3212 | 2973 | 5.47E-10 | 48.9 |
| rps19_len92 | chr2 | 38.667 | 75 | 43 | 1 | 3 | 77 | 38631 | 38416 | 1.48E-11 | 53.1 |
| rps3_len218 | chr1 | 44.898 | 49 | 27 | 0 | 165 | 213 | 98865 | 98719 | 4.30E-08 | 47 |
| rps3_len218 | chr2 | 34.906 | 106 | 65 | 2 | 112 | 213 | 35344 | 35027 | 1.46E-12 | 60.5 |
| rps4_len201 | chr4 | 37.607 | 117 | 61 | 4 | 32 | 141 | 13549 | 13884 | 3.84E-11 | 55.8 |
| rps7_len155 | chr5 | 31.206 | 141 | 87 | 3 | 21 | 155 | 8894 | 8484 | 6.58E-18 | 73.9 |
| ycf1_len1876 | chr3 | 71.014 | 69 | 20 | 0 | 1002 | 1070 | 48775 | 48981 | 1.86E-21 | 96.3 |
| ycf2_len2306 | chr1 | 42.857 | 77 | 33 | 2 | 965 | 1041 | 48143 | 48340 | 1.26E-06 | 47.8 |

**Table S7. Gene sequence of chloroplast transfer to mitochondria in *P. granatum***
